# Recent Progress on Biodegradable Tissue Engineering Scaffolds Prepared by Thermally-Induced Phase Separation (TIPS)

**DOI:** 10.3390/ijms22073504

**Published:** 2021-03-28

**Authors:** Reza Zeinali, Luis J. del Valle, Joan Torras, Jordi Puiggalí

**Affiliations:** 1 Departament d’Enginyeria Química, Universitat Politècnica de Catalunya, Escola d’Enginyeria de Barcelona Est-EEBE, 08019 Barcelona, Spain; luis.javier.del.valle@upc.edu (L.J.d.V.); Joan.Torras@upc.edu (J.T.); 2 Institute for Bioengineering of Catalonia (IBEC), The Barcelona Institute of Science and Technology, c/Baldiri Reixac 10-12, 08028 Barcelona, Spain

**Keywords:** tissue engineering scaffold, thermally induced phase separation, processing parameters, porosity, pore structure, morphology, biodegradable polymer, composites

## Abstract

Porous biodegradable scaffolds provide a physical substrate for cells allowing them to attach, proliferate and guide the formation of new tissues. A variety of techniques have been developed to fabricate tissue engineering (TE) scaffolds, among them the most relevant is the thermally-induced phase separation (TIPS). This technique has been widely used in recent years to fabricate three-dimensional (3D) TE scaffolds. Low production cost, simple experimental procedure and easy processability together with the capability to produce highly porous scaffolds with controllable architecture justify the popularity of TIPS. This paper provides a general overview of the TIPS methodology applied for the preparation of 3D porous TE scaffolds. The recent advances in the fabrication of porous scaffolds through this technique, in terms of technology and material selection, have been reviewed. In addition, how properties can be effectively modified to serve as ideal substrates for specific target cells has been specifically addressed. Additionally, examples are offered with respect to changes of TIPS procedure parameters, the combination of TIPS with other techniques and innovations in polymer or filler selection.

## 1. Introduction

Tissue engineering (TE) is a very tested field of biotechnology that has been developed for over two decades [1,2]. TE aims to construct biological substitutes to restore and maintain normal function of injured or diseased tissues by applying the knowledge of biology, cell transplantation, materials science and bioengineering [3]. In this strategy, a biodegradable three-dimensional (3D) porous scaffold integrated with biological cells or molecules is often studied for regeneration of tissues or organs [4].

3D biodegradable polymer scaffolds with a porous structure usually act as temporary template for seeding, adhesion, growth and proliferation of living cells to guide regeneration and formation of new tissues, while the biodegradable polymer matrix is subjected to biodegradation [5,6]. Moreover, the 3D porous architecture of the scaffold can affect cell migration by regulating the transport of oxygen and nutrients [7,8].

Another important application of biodegradable polymeric scaffold is their use as support materials for different drug loadings. Scaffolds can be designed to allow drug release in a sustained and controlled manner over a desired period of time [9,10].

Various synthetic and natural biomaterials have been widely investigated as scaffolding materials in tissue engineering fields [11]. Among them, aliphatic polyesters such as polylactide (PLA) [12,13,14], polyglycolide (PGA) [15,16], polycaprolactone (PCL) [17,18] and their copolymers like poly(lactide-*co*-glycolide) (PLGA) [19,20,21,22], poly(l-lactide-*co*-caprolactone) (PLCL) [23], poly-(glycolide-*co*-caprolactone (PGCL) [24] and poly(l-lactide-*co*-glycolide-*co*-ε-caprolactone) (PLLGC) [25] have received significant scientific attention due to their good biocompatibility and biodegradability. Furthermore, most of them have been approved by the Food and Drug Administration (FDA) for certain clinical applications [26,27]. Apart from the usage of natural/synthetic polymeric scaffolds in various fields of tissue engineering, a promising strategy is the design and fabrication of binary hybrid/composite matrices consisting of biodegradable polymers and inorganic fillers like hydroxyapatite (HA) [28,29,30,31,32,33] and tricalcium phosphate (TCP) [1,34,35]. These are ideal for regeneration of bone-like tissues.

Many techniques have been developed to produce porous tissue engineering scaffolds, such as porogen leaching [36,37], emulsion freeze drying [38,39], 3D printing [40], gas foaming [41,42], electrospinning [43,44,45], thermally-induced phase separation (TIPS) [46,47] and any possible combinations of two of these techniques [48].

Among all the indicated techniques, TIPS is one of the most efficient due to its ease implementation (i.e., expensive specific equipment are not required) and its potential capability to produce scaffolds with tunable properties [49]. Different parameters can be considered to obtain the required properties for each specific application. The most important are the selection of the polymeric system (including composites), polymer concentration, solvent and nonsolvent system (including ratio between them) and cooling rate [50]. Adjusting such parameters, allows for efficient control over the final structure of the scaffold in terms of morphology, average pore size and degree of interconnected pores, which have a significant and very often limiting role in tissue regeneration [48,51]. Additionally, other important features of scaffolds like biodegradability [52] and mechanical properties [46,47] can also be controlled by the TIPS method. Porous scaffolds of tissue engineering can be prepared by TIPS technique without remnant of solvents, so preserving the biocompatibility of the scaffold [50].

The TIPS method has been developed in 1978 to fabricate microporous membranes [53]. In this process, a homogeneous multicomponent system (polymer, solvent, filler, drug, etc.) under defined circumstances becomes thermodynamically unstable, inducing the system to be separated into two distinct phases [54], a polymer-rich and a polymer-lean phase. After solvent removal by extraction, evaporation or sublimation, the polymer-rich phase is converted into the skeleton of a porous scaffold, while the removed solvent is responsible of the final porosity [55,56]. This method allows obtaining polymeric foams with porosity over 95% [57] and pore diameters from ~1 to 100 µm [53]. Numerous innovations in this area, including combination of TIPS with other fabrication techniques such as electrospinning [58,59], porogen leaching [60,61,62], 3D printing [21,22,63], modification of the solvent removal procedure [14,64] or variations in TIPS parameters [14,21,46,51,64,65] has been reported in literature.

All these advancements have enabled researchers to produce a variety of architectures and pore morphologies in micro/nanometer scale, desirable for specific utilizations. Porous scaffolds with architectures identified as micro/macro-porous [18,22,66], fibrillar (nano/micro-fibrous) [13,18,63,67,68], isotropic (random-pore) [46,51,60,69,70], anisotropic (oriented/aligned-pore or microtubular) [62,64,71,72,73,74], bilayered [58,59,75], biphasic [20,75] structures, lamellar platelets, lamellar stacks, axialites, spherulites [14] or a mixture of these structures have recently been produced by TIPS or TIPS-based strategies.

The great variety of 3D porous polymeric scaffolds developed by TIPS allows to satisfy a wide range of tissue engineering applications for regeneration of cartilage [76], bone [17,60,67,73,77,78], osteochondral [62,72,75], dermal [20], cardiovascular [58,71], neural [59,68,79,80] tissues and so on.

In this paper an overview of TIPS technique is provided and includes essential issues like methodology, influence of processing parameters and explanation of the different mechanisms of phase separation. Recent TIPS and TIPS-based technologies and materials used for fabrication of tissue engineering scaffolds have subsequently been reviewed considering structural features, properties and applications of the scaffolds. In the last section, the paper has been centred on tissue engineering scaffolds with special architectures produced through different mechanisms of phase separation and under specific fabrication conditions.

## 2. TIPS Technique

### 2.1. Methodology

The TIPS technique is based on decreasing the temperature of a homogenous polymer solution or a homogeneous multicomponent system which has been formed at high temperature. The decrease of the thermal energy is used as a driving force to induce phase separation [50]: upon cooling, the system becomes thermodynamically unstable, being induced to separate into two distinct phases [54]. A polymer-rich and a polymer-lean (solvent-rich) phase with high and low polymer concentrations, respectively. The solvent in the polymer-lean phase is subsequently eliminated by extraction, evaporation or sublimation [81]. After solvent removal, the polymer-lean phase forms porosities and the polymer-rich phase is converted into a porous network characterized by appropriate pore geometry and interconnectivity [55,56,64].

**Figure 1:**
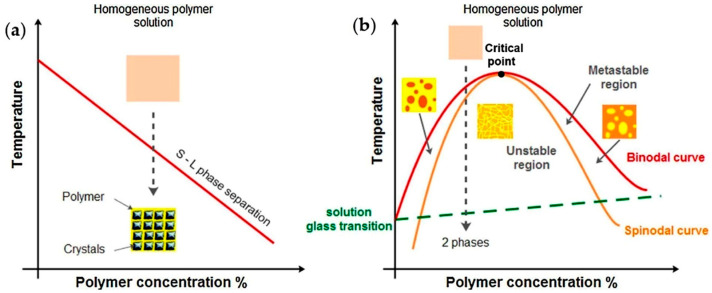
TIPS process in polymer solutions through two main mechanisms: (**a**) solid-liquid phase separation, (**b**) liquid–liquid phase separation. Reprinted with permission from [64].

The TIPS process is a function of polymer-solvent affinity and may follow two typical mechanisms (Figure 1) [64], namely liquid-liquid and solid-liquid phase separations. The former occurs before the solvent freezing, and the latter only happens when the solvent has completely been frozen [53]. The specific way of separation can be selected by choosing the TIPS parameters that affect the thermodynamics of the process (e.g., solvent system, polymer type and concentration, cooling temperature or cooling rate) [64]. Solid-liquid phase separation may happen during cooling when there is a good compatibility between the polymer and solvent (e.g., PLA and 1,4-dioxane with Hansen solubility parameters (HSP) of 21.9 MPa^1/2^ and of 20.5 MPa^1/2^, respectively) [64,82] and the solvent has a relatively high freezing point (Figure 1a). For the occurrence of solid–liquid phase separation in such type of system, the solvent crystallization temperature (freezing point) in the solution must be higher than the liquid–liquid phase separation temperature. By lowering the temperature of the polymer solution, the solvent is crystallized, the polymer is expelled from the crystallization front and the system consequently undergoes solid–liquid phase separation [83]. In this process, the structuration of the network is controlled by the crystallization of the solvent part [53]. Specifically, after removing the solvent, the morphology of the resulting foam is characterized by the pores having a geometry like the solvent crystallites [81,84,85]. For example, in the case of PLA solutions in 1,4-dioxane, a porous structure with an average pore size of around 100 microns has been created [53].

When a polymer-solvent system experiences a solid-liquid phase separation, the final structure and pore architecture of the resulting foam widely depends on the used solvent and its crystallization temperature, the phase separation temperature, the polymer concentration and the temperature gradient which has applied to the system [83]. Typically, an organic solvent with quite high freezing point like benzene (5.5 °C) or 1,4-dioxane (11.8 °C) is used for fabrication of synthetic polymer matrices using solid-liquid phase separation process.

By controlling the cooling process, matrices produced by solid-liquid phase separation may have isotropic or anisotropic architectures. Matrices with a randomly-oriented (isotropic) pore structure are formed by transferring quickly the homogeneous solution to a cooling device that allows solvent crystallization and solid-liquid phase separation. Indeed, when the crystallization direction is controlled and a uniaxial temperature gradient is applied to the system (for example by insulating the side walls of the mould that contains the polymer solution, before transferring it to the cooling device) an oriented-pore structure (anisotropic) is achieved.

Liquid–liquid phase separation in a polymer solution may occur during the cooling process, when the polymer-solvent affinity is very weak. Schugens et al. [86] and Nam and Park [87] showed that by adding water to a PLA/1,4-dioxane system, the phase separation mechanism can be changed under cooling. When water, as a poor solvent of PLA (note that its HSP is 47.8 MPa^1/2^), is added to the system, the interactions between the polymer and solvent are lowered. As a result, a liquid–liquid demixing occurred at the temperatures higher than the solvent crystallization temperature.

The Flory–Huggins equation for the polymer-solvent system is shown in Equation (1) [88]:

(1) $$\frac{\Delta G_{mix}}{RT}=\frac{\phi_{d}}{x_{d}}\ln\phi_{d}+\frac{\phi_{p}}{x_{p}}\ln\phi_{p}+\chi \phi_{d}\phi_{p}$$

where ∆*G_mix_* is the Gibbs free energy of mixing per lattice site, *ϕ _d_* and *ϕ _p_* are the volume fraction of solvent and polymer, *x_d_* and *x_p_* are the number of lattice sites being respectively occupied by the molecules of solvent and polymer, and *χ* is the Flory–Huggins interaction parameter which is affected by the strength of interactions between polymer and solvent.

The first two terms on the right side of the Flory–Huggins equation represent combinatorial entropy contribution and are always negative, while the third term which represents the enthalpic contribution can be positive or negative (in function of the sign of *χ*). Weak interactions between polymer and solvent lead to a large and positive value of *χ*, resulting in a positive ∆*G_mix_* that led to a liquid–liquid demixing. By contrast, when the strength of interactions between polymer and solvent is high (small *χ*), occurrence of liquid–liquid phase separation is more difficult (i.e., lower temperatures are needed). In this situation, the homogenous one-phase region in the phase diagram (Figure 1b) expands, and therefore the binodal curve, which is the boundary of liquid–liquid phase separation, shifts to lower temperatures [89].

In case the solvent crystallization temperature is much lower than the phase separation temperature, the polymer solution undergoes liquid-liquid phase separation upon cooling [83]. Typical phase diagram for a polymer-solvent system, characterized by an upper critical solution temperature (UCST) -critical point- is introduced in Figure 1b [64]. When the temperature is high, the solution is homogenous and in a one-phase region. During cooling, when a homogeneous polymer solution reaches to a temperature-composition point below the binodal curve, a liquid-liquid phase separation occurs, and the system is separated to polymer-rich and polymer-lean phases [83].

According to the thermodynamic pathway (composition, temperature, time), the phase separation can take place with different behaviours [65,87]. The first possible scenario is that demixing occurs below the binodal curve, in a region which is called metastable. In this region solutions are stable with respect to small fluctuations in the composition and there is a kinetic barrier against the phase separation. This kinetic barrier can be overcome by a nucleation process. Hence, the two phases separate through a nucleation and growth (NG) mechanism. This mechanism proceeds by NG of polymer-rich phase in a surrounding matrix of polymer-lean phase, when polymer concentration in the solution is lower than that of the critical point [81]. In this situation a powder-like polymer solid or a bead-like structure is obtained [83,90]. Conversely, NG of polymer–lean droplets in a matrix of polymer rich phase at concentrations higher than critical, results in formation of a foam with closed pores, or a poorly-interconnected polymeric matrix with spherical or pseudo-spherical pores [64,83].

The second scenario which is called spinodal decomposition (SD), can take place when the spinodal curve is crossed for a given polymer concentration under cooling. The region below the spinodal curve is called unstable, where there is no activation barrier against phase separation. Any fluctuations in composition cause free energy to decrease and a wave of fluctuations throughout the solution is triggered. In this region, the system separates into two phases through a well-known SD mechanism, resulting in formation of a bicontinuous and interconnected structure of polymer-rich and polymer-lean phases [81,87,91]. After removing the solvent, a 3D well-interconnected porous network is formed as a result of SD. Generally, the polymeric foams prepared through NG mechanism (metastable region) have relatively large pores while those prepared via SD mechanism (unstable region) have a fine microcellular structure (a network of small interconnected pores) [90,92].

The third possible scenario, which is the most likely one, is a combination of the two previous mechanisms (i.e., NG, and SD mechanisms). This occurs when both curves are crossed upon cooling for a certain polymer concentration. Since liquid-liquid phase separation trough NG is quite slow and through SD is very fast [92], higher cooling rate conditions can be applied to limit or prevent NG mechanism in the metastable region and achieve the fine SD-derived structures [81,89,92,93]. Önder et al., in addition to the cooling rate, by altering other TIPS parameters (i.e., polymer concentration and solvent/nonsolvent ratio) could control the NG/SD-derived morphologies during TIPS process [90]. After the liquid-liquid phase separation through both mechanisms is finished, the resulting structure becomes frozen in a solid state by continuing the cooling process to a temperature below the glass transition or gelation or solvent crystallization temperature in the solution. The next step is the removing of the solvent from the frozen structure to create a polymeric porous network. The conventional method of solvent removal from the phase-separated structures is freeze-drying. This is a long process (i.e., generally takes between three days [39,65,83,87] to one week [94,95]) and, particularly, a high energy-consuming method. Supercritical CO_2_ drying technology is an effective alternative for the reduction of the duration and environmental cost of the whole process that is being successfully developed by scientists [96].

Fabrication of porous matrices via solid–liquid and liquid–liquid phase separations, with the use of organic solvents with high freezing points (i.e., 1,4-dioxane or benzene), and others with low freezing points (i.e., tetrahydrofuran (THF), dimethylformamide (DMF) and pyridine) has been reported [84,85,94,97,98]. Recently, the use of ethylene carbonate (EC) as a solvent for preparation of poly(lactide-*co*-glycolide) (PLGA) foams by TIPS method has also been reported [20,99,100]. This solvent with a melting point of 36.4 °C [101] can be supercooled between 0–10 °C before freezing [102,103]. Furthermore, its high miscibility with water allows its fast leaching from the scaffolds [99], and becomes an appropriate selection when rapid process manipulations are required [20]. Considering the lethal dose (LD50) of 1,4- dioxane, THF and EC, which are 4200–5400 mg∙kg^−1^, 1650 mg∙kg^−1^ and 10,400 mg∙kg^−1^, respectively, EC represents therefore a proper solvent for clinical applications with relatively low health hazards [99,104,105,106].

Porous matrices with the morphology consisting of a continuous isotropic structure with pores ranging from several to tens of microns can be easily produced by means of liquid-liquid phase separation [83]. In some cases, a gelation process may happen during liquid-liquid phase separation upon cooling [107,108]. The formed gel is a network of physically crosslinked polymer chains with trapped solvent within the network [109]. This gelation process have also great interest for fabrication of polymer matrices with nanofibrous structures [83]. In semicrystalline polymers, the interlocking of crystal agglomerates may be the mechanism for the formation of gel [109]. When the gel is formed, the solvent is exchanged and the gel is freeze-dried to render a 3D nanofibrous network.

By utilizing a semicrystalline polymer, due to the crystallization potential of the polymer, the solution encounters driving forces for liquid-liquid phase separation and for crystallization of polymer during cooling. This coexistence somewhat complicates the process. Thus, polymer can crystallize if its crystallization temperature is higher than the phase separation temperature, and also if the solution is held long enough at a temperature higher than that of the phase separation. In this case, another type of solid-liquid phase separation –caused by solidification of the polymer- occurs [83]. This type of phase separation in the literature has also been called “solid-liquid phase separation” and, in some cases, “crystallization-induced phase separation”.

Depending on the polymer concentration, different morphologies can be obtained as a result of crystallization or precipitation of polymer from the solution. These morphologies vary from loose precipitates (unconnected precipitates) to percolating structures (interconnected networks of crystallites) [81]. Specifically, microarchitectures such as platelet-like structures from quite low concentrated solutions [94] and spherulitic structures from relatively high concentrations have been produced [89,110].

As a representative example, it is interesting to note that a semicrystalline polymer like PLA could render a phase separation via a typical liquid-liquid mechanism or via polymer crystallization. As a matter of fact, there is a competition between these two processes due to both mentioned driving forces. When liquid-liquid phase separation precedes the polymer crystallization upon cooling, phase separation occurs through binodal or SD mechanisms. By contrast, if crystallization of polymer precedes liquid-liquid phase separation, the earlier process (polymer crystallization) becomes thermodynamically favourable when the temperature drops below the crystallization point of the polymer. When enough undercooling is available the polymer crystallizes, and the polymer crystals are formed through NG mechanism [111].

### 2.2. Application of Foams Prepared by TIPS

The TIPS technique has been developed to produce microporous membranes in 1978 [53]. This method has widely been used in non-biomedical fields to prepare synthetic membranes for separation and filtration purposes so far. As recent examples of applications of TIPS for nonmedical applications, the production of nanocomposite-based epoxy resins with favourable electrical, thermal and mechanical properties from polyacrylonitrile aerogel and carbon nanofibres (CNFs) can be mentioned [112]. Epoxy resins, as thermosetting polymers with unique physical, mechanical and chemical resistance, can be used in protective coatings, adhesives, high-performance composites, moulding and electrical applications [113,114].

Employing TIPS technique in the biomedical sector is also habitual, for example to develop drug delivery systems. Specifically, this methodology has been used for preparation of microspheres incorporating pharmaceutical and biological agents [3,115]. It should also to be considered among the biomedical sector applications that TIPS is among the common techniques which are employed to manufacture 3D porous scaffolds for tissue engineering applications [25,73,76,80].

A tissue engineering scaffold is a concept of designed and appropriate replacement material for regeneration of tissues such as bone, cartilage, etc. [116]. The scaffold must fulfil essential requirements such as possessing a 3D porous structure with the pores inside and on the surface to allow cell adhesion, proliferation, and differentiation. Such a pore structure also permits the nutrients and waste materials to be transported throughout the scaffold and also helps to form a mimetic host tissue that lead to the regeneration process [117]. Other requirements are biocompatibility, due to degradation of the materials over time [118], adequate mechanical properties until the target tissue is completely regenerated [119] and, in addition, a simple and sustainable manufacturing process [115].

The TIPS method is an effective conventional technique for the fabrication of tissue engineering scaffolds, as it is able to generate highly porous matrices with an interconnected pore network [84,120]. This technique is also compatible with incorporation of high amounts of filler materials, enjoying the advantages of low production cost and easy processability [121]. Specifically, TIPS allows fabricating polymer foams with porosity over 95% [57], with a wide range of pore diameter ranging from ~1 to 100 µm [53]. Due to the all mentioned potentials, and also to the versatility, ease of operation and the vast range of attainable scaffold morphologies, phase separation-based procedures have proven to be reliable methods for creating 3D porous scaffolds [93,122]. TIPS experimentally allows researchers to control the final structure of the scaffold with respect to morphology, average pore size and degree of interconnection [51]. Specifically, it has been reported that process parameters such as the polymer concentration and crystallinity, the cooling temperature and rate, and also the presence of ceramic powders can be manipulated to be able to have control over the indicated morphologic characteristics [84,123].

Depending on the applied polymer and phase separation conditions, polymer scaffolds in terms of structure can be classified as solid-walled isotropic and anisotropic (e.g., microtubular), fibrous, nanofibrous, and platelet-like structures [84,85,94,97,98]. In addition, the different mechanisms of TIPS techniques, including solid–liquid [84,85,97], liquid–liquid [94,98] and crystallization-induced phase separations [94], have been used for the preparation of different micro- and nano-structured polymer networks.

Porosity is a crucial parameter to be noticed when approaching tissue engineering. Numerous attempts have been reported in the literature aimed to manufacture highly porous scaffolds by different techniques and a great variety of materials. For example, PLA-based scaffolds having 20% of β-TCP have been produced by the salt leaching method [124], and composite sponges made of PLGA, collagen (Col) and apatite particles have been prepared by sintering being reached porosity levels so high as 87% [125]. Although there is no consensus about the optimal values concerning porosity and derived morphology in the literature [8], porosity values higher than 90% are suggested for bone tissue engineering applications [124,125,126,127]. This level results in a high cell proliferation, bone ingrowth and osteogenesis [8] as a consequence of the high transport of oxygen and nutrients [128]. TIPS is a technique that without the need of high temperatures (that may affect the polymer matrix or mineral filler properties) allows to prepare scaffolds with porosities higher than 90% [121]. Typical porous architecture produced by the TIPS technique consisted of interconnected pores with a wide range of pore sizes [53], an interesting and desirable feature for tissue regeneration. Thus, larger pores (in tens of microns) allow transport of typical cells and the smaller ones are ideal for perfusion of small molecules such as nutrients and growth factors [129].

Considering the potentiality of TIPS to produce highly porous PCL scaffolds [126], Gandolfi et al. employed this technique to fabricate PCL-based scaffolds loaded with different amounts of calcium silicate (CaSi) and calcium phosphate for bone regeneration. Scaffolds could reach a 95% of open porosity even for a 20 wt% of fillers. The composite scaffolds were not brittle and the inorganic fillers played a reinforcing effect by improving viscoelastic properties. Nucleation of calcium phosphates and apatite on their surface created a bone forming osteoblastic microenvironment [69].

An appropriate bone tissue engineering scaffold allowing cells to grow with appropriate physical shapes and assisting the vascularization of primary tissues, not only needs to have enough porosity, since requires also a well-regulated interconnected pore structure. TIPS is an ideal technique since allows the preparation of 3D scaffolds with adjustable porosity and interconnected pores [130].

Correspondingly, Kozehkonan et al. used TIPS to produce PCL scaffolds with high porosity and pore interconnectivity. As a surface modification to overcome the hydrophobicity of PCL scaffolds, they coated the resulted scaffolds with chitosan (CS), bioactive glass (BG) and nanoparticles of gelatin (GEL). TIPS-obtained PCL scaffolds were highly porous, had variable pore sizes from several to a few hundred microns, and an appropriate pore interconnectivity. The applied surface modification improved the degradation rate and spreading tendency of the cells on the scaffolds. Compared to single or binary coating systems without BG, the proposed ternary system resulted in the highest mechanical strength and a significant improvement in MG-63 cell proliferation [131].

An ideal tissue engineering scaffold to be anatomically adapted must also provide enough mechanical resistance [132]. Montanheiro et al. [74] employed the TIPS technique to produce poly(3-hydroxybutyrate-*co*-3-hydroxyvalerate) (PHBV) scaffolds reinforced with cellulose nanocrystals (CNC). Different levels (1–3 wt%) of nanocrystals were efficiently dispersed by ultrasonication of PHBV/dioxane solutions, and after freezing and freeze-drying steps porous nanocomposite scaffolds were achieved. These scaffolds consisted of oriented pores and unidirectional channels with some regions of random pores. A wide range of pore size distribution was reported: bigger pores in the bulk and smaller ones on the walls which would allow the transport of cells and nutrients. Results revealed that incorporation of CNCs did not affect the morphology but led to a decrease in the porosity of the scaffolds due to changes in the phase separation mechanism. Although they nano-scaled needle-like CNCs were added to the polymer solutions, a dispersion of micron-sized CNC agglomerates was still observed. Therefore, dispersion was not efficient along the whole matrix, especially at higher CNC ratios. However, reinforced scaffolds showed an improvement in the compression modulus and mouse fibroblast cell attachment and proliferation with respect to neat PHBV scaffolds [74].

**Figure 2:**
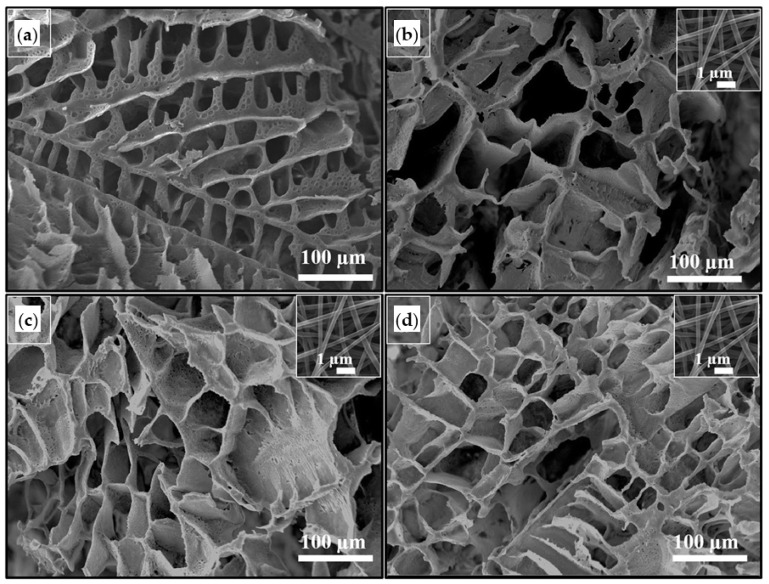
SEM micrograph of the prepared scaffolds. PCL/PLA/GNF (**a**), PCL/PLA/GNF/Tau 0.1% (**b**), PCL/PLA/GNF/Tau 1% (**c**) and PCL/PLA/GNF/Tau 10% (**d**). The insets show SEM micrograph of GNFs. Reprinted with permission from [133].

Taking advantages of structural features of TIPS-obtained scaffolds (i.e., high porosity and interconnective pore structure), and positive biological activities of electrospun GEL nanofibres and taurine, Samadian et al. developed composite scaffolds for bone regeneration. Thus, pre-prepared electrospun GEL nanofibres having different contents of taurine were incorporated to PLA/PCL-based scaffolds. These modifiers were added to a PLA/PCL/dioxane solution and by freezing phase separation was induced. Figure 2 shows the highly porous scaffolds with interconnected porosities that were generated after sublimation of the solvent through freeze-drying. New scaffolds were good candidates for bone regeneration purposes due to their acceptable hydrophilicity, weight loss, mechanical properties, hemo- and cytocompatibility and capability to support bone cell proliferation in vitro and bone regeneration in vivo [133].

**Figure 3:**
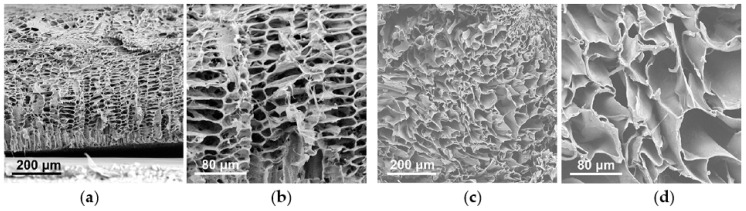
SEM images exhibiting the cross-sectional structure obtained from PCL (**a**,**b**) and PEUU (**c**,**d**) scaffolds prepared using same TIPS experimental conditions (i.e., solvent, polymer concentration, cooling and drying conditions). Reprinted with permission from [66].

In addition to porosity, pore size and structure, biocompatibility of tissue engineering scaffolds must be considered in tissue engineering applications. Presence of organic solvents or inadequate leaching of some porogen agents can affect the biocompatibility of scaffolds. The TIPS process allows fabrication of tissue engineering scaffolds without remaining solvents and, therefore, preserving biocompatibility. Various TIPS-obtained scaffolds with especial characteristics have recently been applied in myocardial, dermal and bone tissue regeneration [20,50,134]. Employing two different synthetic polyesters (i.e., PCL and a poly(urethane urea)ester (PEUU), Wang et al. prepared biocompatible 3D scaffolds by TIPS for massive rotator cuff tear regeneration. An irregular pore structure was obtained from both polymers using the same parameters. An interconnected structure was obtained, being the largest pores about 40 μm and 80 μm for PCL and PEUU scaffolds, respectively (Figure 3). The PEUU scaffold had an ideal biocompatibility, suitable structural properties and elastic mechanical properties. In addition, it could better support the proliferation and migration of rabbit bone (RBMSCs) in vitro and efficiently induced physical tendon-to-bone interface and tendon regeneration in a rabbit model. Hence, the macroporous PEUU scaffold was suggested as an efficient graft for tissue repairing/regeneration [66].

## 3. TIPS and TIPS-Based Technologies for Fabrication of Tissue Engineering Scaffolds

### 3.1. Preparation of TE Scaffolds by the TIPS Technique

**Figure 4:**
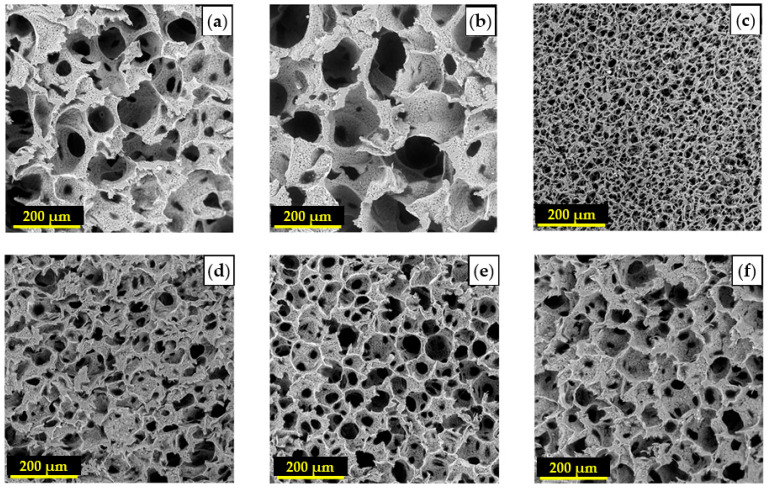
SEM micrographs of PLLA scaffolds obtained by TIPS according to a slow cooling for 30 min and demixing temperatures of 25 °C (**a**) and 30 °C (**b**), and a fast quenching (**c**). The average pore sizes were 100 μm, 150 μm and 25–30 μm, respectively. By keeping the demixing temperature at 20 °C for 15 min and using/not using an insulating PTFE shell (COAT/NOCOAT) in TWB and/or in EAB, (**d**) COAT-NOCOAT; (**e**) NOCOAT-COAT; (**f**) COAT-COAT. The average pore sizes were about 30 μm, 40–50 μm and 60 μm, respectively. Reproduced with permission from [51].

TIPS is one of the most versatile methodologies to produce scaffolds with a high degree of interconnection and a wide range of pore dimensions via targeted temperature versus time protocols [135,136]. Taking into account this capability of TIPS technique, Lombardo et al. [51] controlled porous structure and pore size of poly(L-lactide) (PLLA) scaffolds to be used as 3D support for in vitro culture of tumour cells and studied the effect of porosity and average pore size on cell adhesion and growth. Different demixing temperatures and times (i.e., in a thermal water bath (TWB) of 20–30 C°/15–30 min) were applied to a ternary mixture of polymer-solvent-nonsolvent (i.e., PLLA-dioxane-water). Then the samples were quenched in an ethyl alcohol bath (EAB) at −20 C° for 10 min. By using/not using a polytetrafluoroethylene (PTFE) insulating shell (COAT/NOCOAT) they tuned the heat transfer during the phase separation and/or quenching process and produced 3D PLLA foams with pore sizes ranging from 25 to 150 μm (Figure 4). Demixing temperatures of 25 °C and 30 °C lay within the binodal region, where phase separation occurs through NG mechanism [135] and resulted in average pore size of 100 μm and 150 μm, respectively [51]. Reduction of time and the demixing temperature (i.e., 20 °C) resulted in structures with average pore sizes from 30 μm to 60 μm. In this condition, the lower cooling rate allowed the system to remain for longer time in the metastable region, giving rise to nuclei growth and thus an increase in pore size.

On the other hand, direct quench from 60 °C to −20 °C led to a highly interconnected structure with very small pores of 25–30 μm, which is in accordance with the structure where phase separation occurs through SD mechanism [93,122]. Specifically, the sudden temperature drop caused that the ternary polymer solution mainly remained in the unstable region where the biphasic system was not generated via NG. The two phases interpenetrated each other, resulting in the formation of a scaffold with highly interconnected microporous structure [93]. The work confirms that TIPS is a suitable manufacturing technique for finely tuning the scaffolds architecture. An average pore size of 40–50 μm was obtained being an optimal value for the growth and proliferation of breast cancer cells. Aggregation of tumour cells could be induced and an irregular tumour mass could be formed in vivo [51].

Conoscenti et al. used the ternary solution of PLLA/dioxane/water loaded with various amounts of BG 1393 to produce composite foams via TIPS technique in a single step. They dispersed BG particles in the solution by sonication and applied phase separation by placing the system in a thermal bath (30 °C/75 min) and then freezing it in an EAB. After washing with deionized water and vacuum drying, they produced composite scaffolds and studied their chondrogenesis ability [76]. Functionalization of PLLA with BG1393 took profit of its good processability, low reactivity and the well demonstrated suitability for TIPS processes [137]. Porous PDLLA and BG45S5 composite foams fabricated by TIPS process exhibited anisotropic tubular pore morphology [138], while PLLA/BG1393 composite foams developed a typical TIPS architecture (i.e., highly porous foams with isotropic and an interconnected pore structure in which the morphology was not affected by addition of the BG particles). PLLA/BG1393 scaffolds were considered highly promising for osteochondral articular cartilage repair due to the survival of chondrocytes on the culture period, easy colonization in the inner parts and maintenance of the chondrocyte phenotype [76].

Another advantage of the TIPS technique is the easy incorporation of various desirable materials/agents (depending on the target tissue) to the primary polymer solution. Farzamfar et al. [70] developed PCL/PLA scaffolds containing tetracycline hydrochloride (TCH) antibiotic for bone regeneration. They added different levels of TCH to solutions of PCL/PLA (1:1 *w*/*w*) in 1,4-dioxane and fabricated microporous matrices for local administration of the antibiotic and the evaluation of their bone healing activity. The resulting scaffolds had open microstructures with irregular-shaped pores with diameters around 100 μm and showed antibacterial and osteoinductive properties. The highest in vitro cell proliferation and viability and the highest in vivo bone formation in a rat femoral defect was found in scaffolds having 10 wt% of TCH antibiotic [70].

Although freeze-drying is the most common and the conventional step to remove the organic solvents after the phase separation process, sublimation of the frozen solvent is, relatively, a time- and energy-consuming process. Recently, researchers have paid attention to other effective alternatives to reduce both processing time and energy consumption. Supercritical carbon dioxide (SC-CO_2_) drying technology seems to be an excellent alternative but rarely have been studied so far [96] due to its good extraction ability. Reverchon et al. showed that SC-CO_2_ extraction of 1,4-dioxane from PLA matrices could reduce the residual solvent below 263 ppm after only 4 h, which is in accordance with the concentration limits of 1,4- dioxane (380 ppm) authorized by the pharmacopeial convention [139].

In this regard, some researchers conducted a parametric study of the TIPS process. Thus, by adjusting polymer concentration and molecular weight, solvent miscibility, and cooling temperature, the phase separation of PLA/1,4-dioxane-water solvent system could be finely controlled and, therefore, the structural and mechanical properties of the resulting scaffolds could be tuned. Specifically, PLA scaffolds were prepared using SC-CO_2_ freeze-drying an environmental analysis using the Life Cycle Assessment (LCA) methodology was performed. The combination of TIPS and SC-CO_2_ drying effectively reduced the time and energy consumption of the whole process and 50–90% of the environmental impacts, so application at the industrial scale was potentially feasible [64].

### 3.2. Preparation of TE Scaffolds by Combining TIPS with Other Technologies

TIPS technique has been combined with other TE scaffolds fabrication methods. These more complex processes allowed to meet special requirements for a specific target tissues, to enhance physiochemical, mechanical or morphological properties or to obtain special architectures like hierarchical, biphasic or bilayered structures. Useful information about the components and the structure of some scaffolds fabricated by such combinations are summarized in Table 1. Combination of TIPS with other common methods have been classified and the fabrication process together with recent achievements in this area have been elaborated in the following sections.

#### 3.2.1. Combination of TIPS and Porogen Leaching Technologies

Nowadays, many variations concerning TIPS methodology have been developed by altering process parameters [46,51,65], using different solvents [140] or combining the process with other technologies. One of these variations is focused to increase the pores size of the scaffold by adding porogen particles, which can be removed from the system after freeze-drying in a subsequent leaching step. Sodium chloride [50,140,141,142,143,144] or sugar [145,146] particles are the most common porogens. The increase of pore size through complementing TIPS with the leaching technique led to an improvement in cell proliferation, as for example reported for PLLA scaffolds [146].

Szustakiewicz et al. obtained high-porosity composite scaffolds based on PLLA and HA. Using 1,4-dioxane as solvent, they prepared PLLA solutions loaded with different ratios of synthetic HA powder. After addition of NaCl porogen particles, the samples were frozen and then freeze-dried. Salt leaching was performed by soaking the foams in demineralized water. The composite scaffolds obtained after air and vacuum drying had small pores of up to 50 µm and larger ones up to 400 µm resulting from sublimation of 1,4-dioxane and salt leaching, respectively. The HA content increased PLLA thermal stability and crystallinity (due to nucleation effect of HA). Furthermore, the increase of the HA content increased also the surface hydrophilicity, Young modulus, compression stress of the scaffold and the proliferation rate of pre-osteoblast cells. Although scaffolds were highly porous (i.e., 96–98%), pores collapsed for a 90 wt% of HA. Therefore, a 75 wt% HA content was considered optimum [60].

A porous scaffolds, as synthetic extracellular matrix (ECM), plays an essential role in bone TE since provides a 3D template for cell adhesion and proliferation and also pro-osteoblastic signals for osteoblastic differentiation [147,148,149,150]. Hence, an ideal scaffold for bone tissue engineering must have appropriate mechanical properties, osteoconductivity, and growth factor binding/release capacity for bone formation, to be able to mimic the structure and functions of the native bone matrix [151,152,153,154]. Although significant progress has been achieved in developing biomimetic biomaterials in the last few years, still these requirements are not met in most current 3D scaffolds [155,156,157,158].

To morphologically and chemically mimic the native bone matrix, Yao et al. have recently prepared GEL nanofibrous scaffolds with defined macropore structures, favourable for osteogenic differentiation of stem cells and bone regeneration by employing the combination of TIPS particle and leaching techniques [159,160,161]. Functionalization with disk-shaped nanosilicates with high surface to volume ratio were later performed to improve mechanical properties and osteoconductivity. Specifically, paraffin microspheres (150–300 μm) were selected as porogens and added them to GEL/nanosilicate solutions in ethanol/water (50%). Composite scaffolds had similar morphologies to the pure GEL ones; a hierarchical structure with interconnected macroporous and a Col-like nanofibrous microstructure. Interestingly, the GEL-nanosilicate nanofibrous/macroporous scaffolds could better promote osteoblastic differentiation of human mesenchymal stem cells (MSCs) than the pure Col scaffolds [61].

#### 3.2.2. Combination of TIPS and Electrospinning

**Figure 5:**
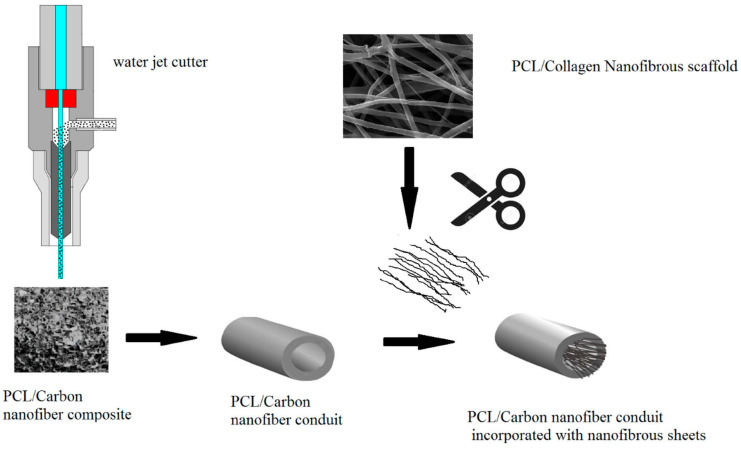
Schematic illustration showing the production process of NGCs. Reproduced with permission from [59].

Scaffolds with structural similarity to natural ECM at the nanoscale and optimum porosity to facilitate metabolites diffusion have been prepared as a neural guidance channel (NGC) by a combination of TIPS and electrospinning methods [162,163]. Scaffolds constituted by electrospun fibres are promising for peripheral nerve repair [164,165], and TIPS is a method with potential to yield pore interconnectivity [166,167]. Incorporation of CNFs to such a complex system, can create electrical conductivity which has been shown that can improve regenerative capacity of NGCs [54]. Accordingly, Farzamfar et al. [59] prepared an NGC (Figure 5) by preparing a PCL scaffold incorporating CNFs by TIPS which subsequently shaped it in the form of a conduit. Thread-like pieces from a PCL/Col nanofibrous sheet (made by electrospinning) were then introduced into the conduit lumen at the time of implantation into a sciatic nerve defect in the rat model. Results demonstrated the potential application of this construct for peripheral nerve tissue engineering since no toxicity or immunogenic reactions were detected, while successful nerve regeneration was well supported [59].

Due to their good biocompatibility, natural polymers are regularly used for the preparation of vascular scaffolds [168]. Nevertheless, tensile strength of such scaffolds is much lower than that of the human coronary artery [169,170,171,172,173]. In order to prepare scaffolds with sufficient mechanical properties to be used as vascular graft, Guo et al. fabricated bilayered small diameter tubular scaffold by combining TIPS and electrospinning. As an inner layer, a microporous GEL foam incorporating 10 wt% of salvianic acid (SA) loaded-mesoporous silica nanoparticles (MSNs) was firstly prepared using TIPS technique. SA was used as drug anticoagulant. In order to strengthen the vascular scaffold, electrospun poly(ester-urethane)urea (PEEUU) nanofibres were deposited outside of the inner layer. The final material showed good mechanical properties, a sustained release profile, a good proliferation of endothelial cells and in vitro long-term anticoagulant efficacy. In addition, hyperplasia and thrombosis effects were not detected in the rabbits’ carotid arteries [58].

#### 3.2.3. Combination of TIPS and 3D Printing

Tissues appropriate for bone regeneration are problematic considering the bone structure, which is a multilevel complex system composed of inorganic and organic components, and the bone formation, which depends on the micro/nano-scale hierarchical structure of ECM [174]. Accordingly, micro/nano-fibre combined scaffolds appear advantageous since can exhibit better cell adhesion and angiogenic potential compared to typical micro-fibre scaffold. Note that an ECM-like nanofibre network has a higher available cell adhesion area and a blood vessel-like structure throughout the formed scaffold [175]. Hence, preparation of multifunctional scaffolds with biomimetic ECM hierarchical structure can be a promising advancement in the treatment of bone defects.

Since TIPS is one of the techniques which are used to construct scaffolds with nano-sized structures [176], combining it with a technique like 3D printing can hopefully overcome the shortcomings of a single technology to get complementary advantages. In this regard, some researchers concluded that 3D-printed PLLA scaffolds having only micro-sized fibres and large-sized pores cannot match the ECM-like hierarchical structures. Therefore, these scaffolds are not particularly conducive to cell adhesion and internal migration that are needed for the formation of high-density vascular networks and bone tissue [177].

**Figure 6:**
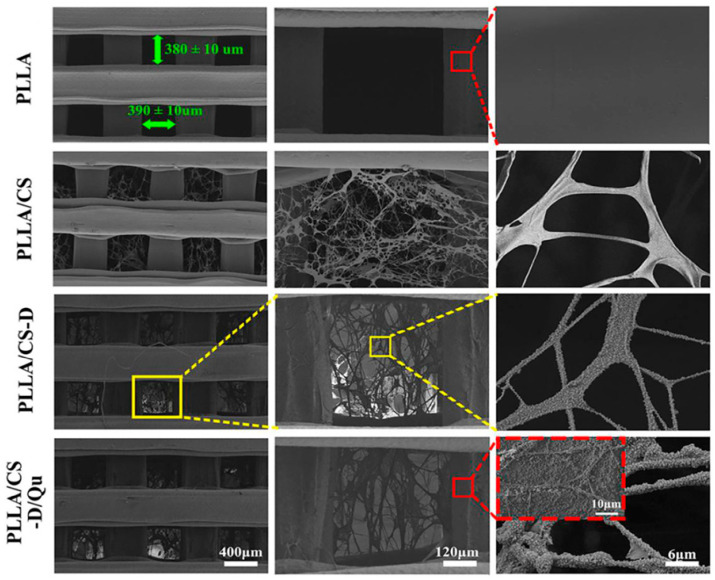
FESEM images of unmodified and modified PLLA scaffolds. The original 3D printed PLLA scaffold had square-like porosity with fibre diameters around 410 ± 18 μm. The transverse distance and the longitudinal distance between two fibres were 390 ± 10 μm and 380 ± 10 μm, respectively. Many particles appeared on the fibre surfaces after successive functionalization with PDA and Qu. Reproduced with permission from [63].

However, combination of 3D printing and TIPS technologies can overcome the indicated limitations. Specifically, through TIPS technique, scaffolds consisting of CS nanofibres within 3D printed PLLA microfibres were produced. The resulting hierarchical structure consisted on irregular CS nanofibres, with diameters around 80–600 nm, which were infiltrated into the holes and the surfaces of the PLLA microfibres. These PLLA/CS scaffolds were finally covered with a polydopamine (PDA) layer (PLLA/CS-D) and then functionalized with bioactive quercetin (Qu) (PLLA/CS-D/Qu). The CS nanofibres were not structurally affected by PDA and Qu surface modification (D/Qu) and maintained their original structure. Field emission scanning electron microscope (FESEM) images of unmodified and modified PLLA scaffolds are shown in Figure 6. Incorporation of CS nanofibres, PDA and Qu increased the hydrophilicity and mechanical properties of the neat PLLA scaffold and promoted cell growth and adhesion into the internal pores. Furthermore, an excellent osteogenic activity and anti-inflammatory response conferred an attractive prospect for bone tissue engineering [63].

The necessity of highly-porous networks for cell seeding and tissue growth, complicate the preparation of high-module scaffolds suitable for bone tissue engineering. Some investigators presented the design optimization of PLGA/nanohydroxyapatite (nHA) scaffolds, prepared by TIPS. By applying different experimental parameters including TIPS temperature, PLGA concentration and nHA content, scaffolds with different thickness, density, porosity, and modulus were produced [21]. The possibility of creating macro/microporous architectures was also considered due to the role of macrochannels in transport, and therefore in cell migration and angiogenesis upon in vivo implantation. To this end, extrusion-based additive manufacturing (AM), (i.e., 3D printing) and TIPS techniques were combined.

**Figure 7:**
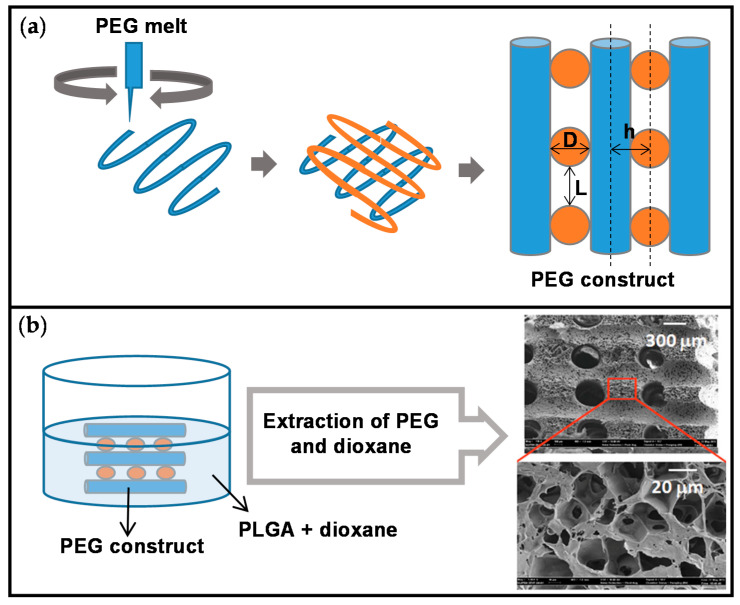
AM bioplotting of PEG constructs and adjustable bioplotting parameters (**a**). Schematics of the fabrication process of a simple PLGA scaffold derived from the previous incorporation of PEG macrofibres (**b**). D,L and h represent strand diameter, pore size and layer thickness, respectively. The successive PEG layers (blue and orange) have perpendicularly been 3D-plotted. Based on [120].

A 3D-plotted strand structure of polyethylene glycol (PEG) was embedded in PLGA/nHA/1,4-dioxane solution and then the system was cooled to get phase separation. In this way a microporous matrix surrounding the PEG macrofibres was obtained after dioxane sublimation. Deionized water extraction of PEG led to the formation of macrochannels [22]. The produced hierarchical PLGA scaffolds with the same procedure had a bimodal pore size distribution (<50 and >300 μm) containing interconnected macrochannels (Figure 7). The orthogonally interconnected channels generated by AM could provide an ideal environment for bone ingrowth, as well as facilitate the transport of oxygen and nutrients. These macro/microporous scaffolds had a potential to act as support materials for in vitro MC3T3-E1 cells growth [120].

#### 3.2.4. Combination of TIPS and Textile Technology

Although textile meshes due to impressive mechanical and manufacturing properties are potential scaffolding materials, both biological and mechanical factors must be considered when designing and manufacturing scaffolds for tissue engineering [20].

Knit mesh structures with tunable tensile properties and compatibility with suturing to adjacent tissues are ideal for dermal tissue repair, but the presence of large void spaces between the fibre bundles complicates the formation of a continuous epithelial cell layer [178,179]. Equipping strong biodegradable textile meshes with a finer secondary structure, which is able to connect fibre bundles and provide a support for cell delivery and growth, is a reliable solution for dermal and other tissue repairing applications [20].

**Figure 8:**
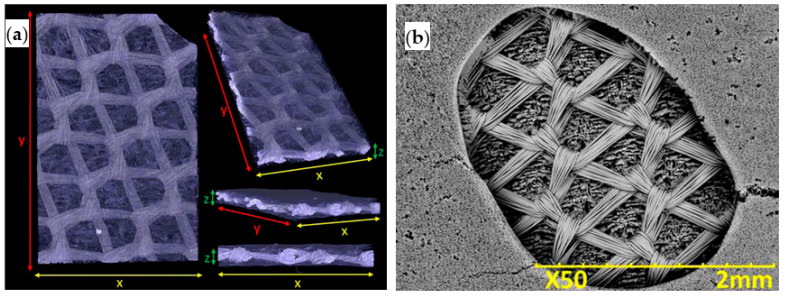
Micro-computed tomography (MicroCT) images of a TIPS-obtained PLGA scaffold incorporating PLGA knitted mesh (**a**) and a SEM image showing the porous TIPS structure and the embedded PLGA mesh through a rare spontaneous bubble (**b**). *X*, *Y* and *Z* are the scaffold dimensions. Reproduced with permission from [20].

Biphasic scaffolds consisting of PLGA textile knitted meshes embedded in a TIPS-obtained PLGA porous structure have for example been developed. Specifically, PLGA strips were immersed in PLGA/EC solutions and quenched to 4 °C. Phase separation was induced by agitation of the supercooled solution and the solvent was immediately leached in Milli-Q water. Utilizing EC solvent was beneficious since facilitated rapid manipulation and minimized the risk of degradation of PLGA meshes. Integrity and mechanical properties of pre-fabricated textile PLGA meshes was preserved after processing and their tensile strength was unchanged. The microstructure of the resulting biphasic scaffolds consisted of the polymer mesh uniformly encapsulated within an interconnected highly porous TIPS structure with an average pore size of 16.85 ± 3.8 μm (Figure 8) [20].

## 4. Materials Used in Fabrication of Tissue Engineering Scaffolds by the TIPS Technique

### 4.1. Polymeric (Synthetic, Natural and Blended) Scaffolds

With the rapid progress in the field of tissue engineering, a great variety of scaffolding materials have been developed, being effectively used as biomedical materials in the treatment of tissue defects and the alleviation of the suffering of patients [180,181]. Various scaffolds made of synthetic and natural biomaterials have been broadly investigated and used in tissue engineering fields, opening up the world of organ and tissue regeneration [11]. Since biodegradability is a key factor for a tissue engineering scaffold (because of obviating the need for removing the scaffold when healing occurs) [25], biodegradable polymers have been extensively utilized in the construction of tissue engineering scaffolds [182]. Among them, aliphatic polyesters, such as PLA, PLGA, PCL and PLCL, have been closely studied over past decades, due to properties like biodegradability and biocompatibility [183]. Having such properties, and also good mechanical properties, PLA has been widely used in TIPS technique to fabricate porous scaffolds of tissue engineering [184,185].

Chen et al. synthesized PLLGC terpolyester by melt copolymerization process and then utilized it for preparation of porous scaffolds by TIPS technique. By varying some TIPS processing parameters like polymer concentration (from 2.5 to 15 wt%) and freezing temperature (from 4 to −60 °C) structural and mechanical properties could be controlled. Results showed larger pores and higher porosity, but a lower compressive strength when the polymer concentration was decreased, or the freezing temperature was increased. Furthermore, regular and interconnected pore structures with uniform pore size was achieved, although at high polymer concentration some pores were linear with uneven distribution and inconsistent pore sizes. The decrease in the freezing temperature caused a structural change from an irregular pore distribution to an orientated micro-tubular structure. Changes could be justified considering the crystallization of the employed 1,4-dioxane solvent throughout the phase separation process and the influence of the temperature gradient, which is more significant at lower freezing temperatures [25].

Materials currently employed in tissue engineering are mainly derived from natural polymers and synthetic materials. Natural-based polymers such as alginate (Alg), GEL, gelatin methacryloyl (GelMA), hyaluronic acid (HAc), CS, Col, fibrin, and decellularized ECM, play an important role in the field of biofabrication. Natural polymers generally enjoy considerable advantages in terms of biocompatibility, biodegradability and supporting bioactivity [186]. Additionally, naturally- or biologically-derived materials are also used due to the general lack of synthetic biomaterials responding to physiological stimuli [187].

Col as a kind of ECM proteins plays an important role in supporting cell attachment, migration and proliferation, due to its excellent biocompatibility, biodegradability and low immunogenicity [188]. Due to such properties, it has widely been used to prepare scaffolds for tissue engineering and regenerative medicine [189]. CS is another natural polymer with good properties such as antibacterial action, hemostasis, mechanics and cytocompatibility which has also been used in a variety of biomedical fields including wound healing, drug delivery carriers, surgical thread and tissue engineering [190]. Naturally-based polymers with their improved biocompatibility are notably employed to fabricate tissue engineering scaffolds using phase-separation technologies [91].

**Figure 9:**
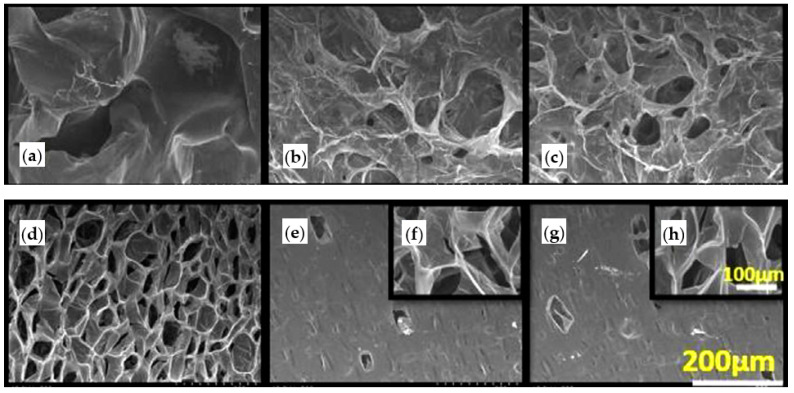
SEM morphologic observations of pure Col (**a**), pure CS (**g**,**h**) and CS/Col scaffolds with wt% ratios of: 10/90 (**b**), 50/50 (**c**), 70/30 (**d**) and 90/10 (**e**,**f**), respectively. All micrographs have the same magnification as well the two insets. Reproduced with permission from [79].

Si et al. [79] fabricated for example porous CS/Col scaffolds with controlled degradation behaviour by TIPS. These natural-based blend polymer scaffolds were employed as a potential support matrix for peripheral nerve regeneration. Different polymer solutions with different volume ratios (ranging from 10–90%) of CS and Col in acetic acid, and pure CS and Col solutions as control were processed. Pore size, degradation rate and mechanical properties of the resulting scaffolds could be tuned by changing the ratio of the two components. Thus, the increase in CS content led to a decrease of the mean pore size, liquid uptake and degradation rate, while improved mechanical properties. Morphological evaluations showed fibre-like structure with an interconnected network in pure Col scaffolds and those with low CS content, while this structure gradually disappeared and changed into a cobblestone-like structure when the CS content reached 90% (Figure 9).

The prepared scaffolds logically showed good cytocompatibility to L929 fibroblasts, lack of toxicity and great capability to promote the attachment, migration and proliferation of Schwann cells. A modulated degradation behaviour without any inflammatory reaction was found when the Col/CS scaffolds were subcutaneously implanted in rabbits, indicating the potential application of these TIPS-obtained and natural-based scaffolds in tissue engineering [79].

Although biomaterials from both synthetic or natural resources are potential candidates for tissue engineering applications, the synthetic ones can be better modified in terms of geometric structure, biocompatibility, porosity and mechanical properties [191]. However, low biodegradation rate, hydrophobicity, and lack of cell recognition sites limit tissue engineering applications of a synthetic biodegradable polymer like PLLA. Thus, blending synthetic polymers with other natural polymers could be an alternative solution [192]. For instance, blending with CS is considered as an efficient approach to obviate some of the indicated drawbacks [193]. Such blending permits taking advantages of both polymers not only as scaffolds for bone [194], cartilage [195] and nerve [196] tissue engineering applications, but also as drug delivery carriers [197].

TIPS appears an advantageous technique since it is highly flexible to blend the polymer-solvent system with other polymers, solvents or inorganic fillers for specific purposes. Salehi et al. employed solid-liquid phase separation to produce porous scaffolds from PLLA and CS. Such blending was aimed to mimic cellular microenvironment and anatomical structure of nerve tissue. The scaffold behaviour and properties were compared with those made from pure PLLA or CS. CS/acetic acid:water (50:50) solution was specifically added to a PLLA/chloroform one, and then solid-liquid phase separation was induced by quenching the mixture to −80 °C (i.e., a lower temperature than the freezing point of both solvents, 63.5 °C and 16 °C for chloroform and acetic acid, respectively). After freeze-drying the resulting scaffolds were soaked in glutaraldehyde to cross-link the CS. The blend scaffolds were highly porous with interconnected porosities and showed proper mechanical properties. In addition, they were more porous and hydrophilic, and had higher degradation rate and lower amounts of free radicals compared to neat PLLA. Furthermore, the blended scaffold could better support human nerve cells than pure PLLA and CS scaffolds, indicating than an effective strategy to prepare promising scaffolds for nerve tissue engineering applications was developed [80].

### 4.2. Composite (Natural/Synthetic Polymer-Ceramic) Scaffolds

Bone is a hard tissue that acts a supporting framework or skeleton of the body and protects organs [198]. Bone tissue regularly heals with minimal scarring, but critical-sized bone defects fail to heal naturally [199,200]. Effective grafting techniques for treating bone defects, i.e., bone autografts and allografts often have problems associated with insufficient bone cell availability, donor site morbidity and bone transplantation failures [201]. Thus, there is a growing need for effective synthetic bone substitute materials [202]. Bone substitutes made of conventional bioceramics, such as HA and beta tricalcium phosphate (β-TCP), have made a significant contribution in orthopedic applications to repair and augment the damaged bone tissues [203,204,205]. Tissue engineering is an effective therapy for bone defects which utilizes scaffolds, cells and growth factors to fabricate artificial bones to be implanted into patients [206]. Taking into account that natural ECM is a composite consisting of naturally occurring biopolymers (organic) and biological apatite bioceramics (inorganic) with a well-oriented crystalline structure, the important aspect for developing an appropriate bone scaffold is to get a suitable 3D porous composite with the specific characteristics to mimic the ECM [207,208,209]. Therefore, an effective material selection requires to combine biodegradable polymers able to be biologically degraded and replaced by growing cells and inorganic bioactive particles [210,211]. A practical approach to enhance cell growth and adhesion and also to avoid the implant rejection is to provide the growing cells with supporting materials with both suitable structure and chemical-physical properties [212,213]. Porosity, and a well-regulated interconnected porosity, are crucial factors for designing scaffolds to mimic the bone architecture [5,214]. In contrast with other conventional methods such as salt leaching, gas foaming and freeze drying, TIPS has proven to be capable of developing interconnected microporous structures similar to the bone architecture [215,216].

Collagen type 1 (Col1) and HA are the major constituents of human natural bone tissue and consequently have widely been studied as promising materials for scaffold preparation. Thus, Wang et al. reported the usefulness of porous Col1/nHA scaffolds for restoration of critical-size bone defects [217]. Bioactive molecules like Col1 and CS have also been used as composite components for regeneration of bone tissue [218,219,220,221,222].

Reves et al. recommended pore sizes of 100–800 µm for microparticle-based scaffolds [223], being the recommended porosity to accommodate osteoblasts or osteoprogenitor cells ≥90% [224]. In order to meet such requirements, Rahman et al. chose TIPS technique as the methodology to develop composite HA/Col/CS scaffolds for restoration of defected maxillofacial mandible bone, using different cross-linkers. Irregular 3D porous microstructures with 3D interconnected fibre microstructure, were obtained, while the pore diameter was ranging from 98 to 204 µm and the porosity reached the recommended porosity of ≥90%. Scaffolds showed desirable blood biocompatibility without cytotoxicity. Scaffolds could also be cross-linked by either irradiation or de-hydrothermal treatment. Final materials were compatible for MSCs attachment and growth, mineralization and supported in vivo new bone formation in rabbit model [225].

Osteochondral tissue is comprised of an articular cartilage region and a subchondral bone region, while a calcified cartilage connects these two microenvironments [226]. Multiphasic scaffolds, in which different layers can model different microenvironments, are the basis of new potential treatment approach. However, the achievement of a stable joining between layers during fabrication remains challenging [75]. Osteochondral tissue regeneration in monophasic scaffolds is lower than multiphasic ones, and due to inadequate mimicking of the native microenvironment they are unable to simultaneously support chondrogenesis and osteogenesis [227,228]. Accordingly, Erickson et al. using TIPS developed multiphasic bilayered scaffolds that were able modelling native osteochondral by combining two distinct scaffolds optimized for cartilage and bone, respectively. Polysaccharide and glycosaminoglyca-based scaffolds were obtained using a mechanical stiffness gradient. Specifically, scaffolds consisted of a CS/HAc layer (as cartilage part) and a Cs/Alg/HA layer with higher stiffness and osteoconductivity (as bone part). The bilayered scaffolds showed seamless gradient transition zone at the interface with increasing stiffness and calcium content resembling that of native osteochondral tissue. Osteogenesis and chondrogenesis were found in the associated layers during a two-week co-culture with chondrocyte-like (or MSCs) and osteoblast-like cells and, importantly, showed the potential of TIPS for fabrication of multilayered scaffolds for osteochondral TE as well as for other complex layered tissues [75].

Binary hybrid materials made of biodegradable and bioresorbable polymers such as PGA, PLLA [229], poly (D,L-lactide) P(DLLA), PCL or their copolymers [230] have been widely studied for bone tissue engineering or regeneration of damaged bone. As polymer fillers, inorganic materials like TCP [1,34,35], HA [28,29,30,31,32,33], bioglass [231,232] and FeHA (Fe-doped nHA) [233,234] are generally used.

Díaz et al. [12] produced nHA/PLLA composite scaffolds and films using TIPS technique and solvent casting method, respectively. The 10–50 wt% of nHA were added to 2.5 *w*/*v*% PLLA/chloroform solutions and dispersed through ultrasonic stirring. This nanocomposite system was subsequently used for fabrication of such scaffolds and films. Thermal and mechanical properties of prepared composite scaffolds and films during the in vitro degradation period was evaluated and the influence of nHA content on properties and structure was determined. TIPS scaffolds showed a highly porous structure, while films had a dense and non-porous structure. An increase in elastic modulus and yield stress was characteristic for all samples when nHA particles were incorporated, but these parameters decreased faster in the film samples than scaffolds during degradation. Films also degraded more rapidly in a heterogenous way due to an autocatalytic effect caused by more difficult release of acidic degradation products. The improvement of mechanical performance was scarce for high HA contents (i.e., 50 wt%) due to agglomeration effects and the corresponding reduction of the interfacial contact with the polymer matrix [12].

Particle dispersion in the polymeric matrix is a parameter with crucial importance and that must be controlled to obtain homogeneous nanocomposite materials. Achievement of a good dispersion is still one of the main problems when preparing composites through mechanical stirring or ultrasound mixing methods. “In situ synthesis” of nanoparticles within the polymer matrix is an efficient alternative to the conventional mechanical methods of particle dispersion, enabling to overcome the strong agglomeration trend of nanoparticles which leads to form micrometric aggregates. In this method, fillers are directly synthesized in the hosting polymeric matrix, hence, particle aggregation is prevented and dispersion is improved [235]. Interestingly, this synthetic method is particularly compatible with the TIPS technique allowing in addition the fabrication of porous structures [121,236]. Recently, combination of in situ synthesis and TIPS for manufacturing PLA-based nanocomposite scaffolds containing HA and polyaniline (PANI) nanoparticles have been reported [67,77].

**Figure 10:**
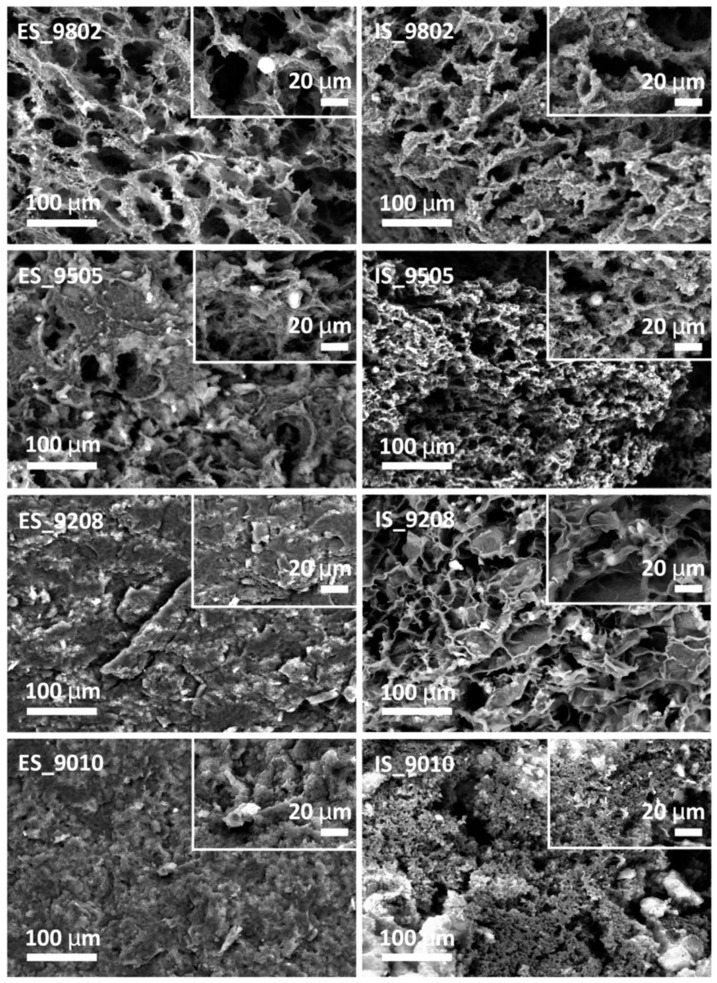
Cross-section SEM micrographs of PHB/HA composite scaffolds. PHB scaffolds containing 2–10 wt% of ex situ- and in situ-synthesized HA nanoparticles have been indicated as (ES9802 to ES9010) and (IS9802 to IS9010), respectively. Insets show higher magnification for each sample. Reprinted with permission from [70].

In a recent study, Esposti et al. [77] produced nanostructured composite porous scaffolds based on poly(3-hydroxybutyrate) (PHB). Composite suspensions were prepared according to two different methods: The innovative in situ synthesis of HA nanoparticles in PHB/dioxane solution, and the conventional mechanical dispersion of ex situ synthesized HA nanoparticles in the same polymer solution. By applying TIPS to all suspensions in addition to solvent exchange using ethanol and vacuum drying, two groups of porous scaffolds were obtained and compared. In situ synthesis of HA clearly limited the particle aggregation problem and rendered high porosity even at relatively high HA contents (up to 8 wt%), while the scaffolds containing ex situ synthetized HA particles were not porous at high HA contents (Figure 10).

Although both scaffold groups inhibited pre-osteoblast cell adhesion and proliferation, and could promote osteogenesis, the scaffolds loaded with in situ-synthesized HA produced the highest amount of alkaline phosphatase and the typical morphology of the terminal differentiation stages of osteoblasts. These findings justify extending this new TIPS-based fabrication process to other polymeric matrices or inorganic nanoparticles, as a simple and scalable tool for the preparation of high surface area polymer-based composites with tailored properties [77].

Pure PLA still has some deficiencies to be used as a biomaterial in bone tissue engineering. Bone tissue engineering puts a great demand on mechanical properties. However, among all PLA deficiencies for its right application in bone tissue engineering, it should be emphasized its moderate strength makes it an unsuitable choice for bone fixation at the site of loadbearing [237], and also its strong hydrophobicity that is not conducive to cell adsorption and growth [238]. To overcome such problems a filler material like CNC could be helpful, due to its strong hydrophilicity and excellent mechanical properties [239,240,241]. In a recent approach Luo et al. fabricated PLA-based scaffolds reinforced with CNC by combining in situ polymerization and TIPS technique. In this approach, CNCs were firstly prepared by acid hydrolysis of cellulose fibres and then nanocomposites were obtained through in situ polymerization of lactic acid and CNCs. The resulting nanocomposites were dissolved in a dioxane/water system to apply the typical TIPS process. Final scaffolds showed higher compression modulus, hemocompatibility, protein adsorption in vitro than neat PLA scaffolds. The proposed nanocomposite scaffolds were also promising for bone tissue engineering considering their good mechanical properties, biocompatibility, biomineralization and bioactivity [242].

Polyester-based scaffolds reinforced with multiwalled carbon nanotubes (MWCNTs) have recently been produced by TIPS [19,243,244]. Possessing a wide variety of properties such as electrical, thermal, mechanical and structural, MWCNTs have been reported as proper candidates for biomedical applications [245]. Even presence of small quantities of carbon nanotubes (e.g., <0.5 wt%) in a polymer matrix can lead to a significant increase in mechanical properties. Note that the average Young modulus of native human bone, bioglass and HA are 12–18 GPa, 35 GPa and 95 GPa, respectively, while that of the MWCNTs is between 200 to 1950 GPa [246]. Apart from mechanical properties, choosing CNTs as filler materials was especially due to their magnetic properties, which enable scaffolds to guide the proliferation, differentiation and mineralization of bone cells [247].

Díaz et al. [19] incorporated small amounts of MWCNT fillers into PLGA-based scaffolds. Nanotubes were dispersed in a PLGA-dioxane solution by ultrasonication and the final composite scaffold was obtained by the TIPS method. Ultrasonic dispersion and TIPS technique facilitated the incorporation, dispersion and distribution of CNTs in the polymer matrix. In addition, TIPS allowed a good control over morphology and porosity of the composite scaffolds.

They reported that the addition of small quantities of carbon nanotubes (0.1–0.5 wt%) to PLGA scaffold could modify in vitro degradation, magnetic properties and cytotoxicity of the scaffolds [19]. The effect of higher levels of MWCNTs (i.e., 1–10 wt%) and the incorporation of HA (i.e., 10 wt%) on PCL-based scaffold properties was subsequently evaluated. Samples were obtained by ultrasonic dispersion of MWCNTs and nHA particles in PCL-dioxane solutions followed by TIPS to get porous composite matrices [243]. Despite the excellent properties of CNTs, having other features like high surface area, small diameter and high aspect ratio (>1000) predispose them to form agglomerates when being incorporated as reinforcements into biopolymers, especially in high loading levels [248]. They reported that both mechanical properties and in vitro degradation were clearly modified by adding MWCNTs: up to 5% of MWCNTs the structural arrangement of the scaffolds was positively changed, but higher levels led to agglomeration, an irregular structure and a negative effect on mechanical properties [243]. The decrease of nHA content to 1 wt%, allowed a clear evaluation of the influence of various levels of MWCNTs (1–10 wt%). Although mechanical properties and conductivity of these scaffolds were improved by adding MWCNTs, properties were dependent on the concentration of nanotubes. Thus, PLLA scaffolds with up to 5 wt% of nanotubes had the best properties with very uniform and a well-arranged structure, but at a higher nanotube concentration scaffold properties dropped due to aggregation, indicating that ultra-sonication/TIPS techniques were not suitable for production of composite scaffolds with such high levels of fillers [244].

Gandolfi et al. [121] tried to incorporate different loading levels of dicalcium phosphate dihydrate (DCPD) and/or hydraulic CaSi into PLA-based scaffolds using TIPS. The resulting composite scaffolds were highly porous (i.e., >90%), biocompatible and bioactive. Furthermore, the presence of the added minerals helped to overcome typical PLA disadvantages such as hydrophobicity, release of acid degradation products and reduced cell adhesion and growth [121]. Bone regeneration ability was subsequently improved by enriching these composite scaffolds with exosome vesicles (EVs) that were derived from MSCs and deposited on the scaffold surface. EVs are released from all cell types and are able to modify the activities of target cells by proteins, growth factors, mRNAs, and micro RNAs internalization, facilitating in addition cells communication [249]. Exosome-enriched composite scaffolds, especially those with the highest amount of mineral fillers could properly stimulate osteogenic commitment of human adipose mesenchymal stem cells (hAD-MSCs). Therefore, new scaffolds could be considered to have a promising potential in regenerative bone healing using stem cells [78].

PCL scaffolds containing different ratios of micro-fluorcanasite (μFC) glass-ceramic particulates, as potential bioactive reinforcements, have recently been prepared by TIPS. Chloroform was selected as polymer solvent, ultrasonication was applied to disperse glass-ceramic particulates in the polymer solution, and liquid nitrogen to freeze the samples and induce phase separation. All the produced matrices revealed porous cellular microarchitectures. The interconnected microporous structure was enhanced with high amounts (e.g., 30 wt%) of μFC glass-ceramic particulates, a feature that attributed to a possible nucleating effect of particulates. A tunable biodegradation was also found since the increase of glass-ceramic content resulted in a delayed biodegradation of PCL matrices (i.e., structural integrity was enhanced upon incorporation of crystalline μFC particles). Owing to favourable microporous architecture and tunable degradation, PCL/μFC biocomposite scaffolds appear as potential matrices for bone tissue engineering [17].

Developing innovative ceramic fillers in bioengineering, TiO_2_ has been identified as an effective biological material because the presence of surface OH groups induces formation of HA from simulated body fluid (SBF) [250]. However, in vitro toxicity inducing pulmonary inflammatory response has been reported for TiO_2_ nanoparticles [251]. Functionalization is probably one of the most effective strategies to prevent this toxicity and enhance the stability of nanofillers in certain polymer matrices [252]. Buzarovska et al. functionalized the surface of TiO_2_ nanoparticles with oleic acid (OA) and prepared PLLA nanocomposite scaffolds by means of the TIPS technique. Microporous structures were obtained after homogenizing the nanocomposite PLLA/TiO_2_/dioxane suspensions by ultrasonication and applying the typical TIPS process. In terms of morphology, a highly porous and anisotropic architecture with characteristic interconnected and elongated pores (length of 70–150 µm, wall thickness of 7–12 µm) was produced. The pore architecture was not influenced by the incorporation of nanoparticles, but nice and evenly distributed nanoparticles were observed as small white dots. The effect of incorporating functionalized nanoparticles on biodegradability, bioactivity and cytocompatibility was also evaluated. Interestingly, scaffolds were bioactive in supersaturated fluids, but became less degradable in simulated body fluid (SBF) than pure PLLA scaffolds. Adding 5 wt% (with respect to the polymer content) of OA-functionalized TiO_2_ nanoparticles enhanced cell viability and proliferation, but the increase to 10 wt% led to worse cell proliferation and higher cytotoxicity [73].

## 5. Architectures Obtained through the Manipulation of the TIPS Process Conditions

Highly porous interconnective network with isotropic or randomly-oriented pore structure can be obtained under different thermodynamic and kinetic conditions of phase separation (e.g., through liquid-liquid and solid-liquid processes) as detailed in the Section 2.1. It has also been explained how by altering TIPS parameters the morphology of the resulting foams can be controlled. However, by applying especial conditions in TIPS process (e.g., controlling temperature gradient, crystallization direction during solid-liquid phase separation or gelation temperature during liquid-liquid phase separation) other types of architectures can be achieved. In addition, by employing a semicrystalline polymer when polymer crystallization precedes liquid-liquid phase separation during cooling, new morphologies arising from the crystallization of the polymer matrix can be produced. In the next sections some related advances and recent studies focused on producing such structures are reviewed.

### 5.1. Nanofibrous Structures Prepared by Liquid-Liquid Phase Separation

ECM is the ideal 3D template for cell adhesion, proliferation and differentiation and is the reservoir for many growth factors [253,254]. Most components of natural ECM are organized in vivo according to a nano-scale fibrillar construction [13]. Nanofibrous ultrastructure of native ECM provides topological cues for specific cellular behaviour and, therefore, this complex architecture could be a good pattern for design and fabrication of bio-scaffolds [68].

Several techniques have recently been investigated so far to prepare 3D nanofibrous scaffolds, including electrospinning [255,256,257,258], self-assembly [259], thermally-induced phase separation (TIPS) combined the porogen leaching technique [61] and 3D printing [260]. The current techniques for producing biomimetic nanofibrous scaffolds have some limitations, for instance, a significant challenge for most 3D printers is to print nanofibrous structures in a well-controlled manner that demand very long processing time and high-end and expensive instruments. These limitations drove investigators to develop an innovative and facile technique for this purpose [18]. TIPS has been favoured by many researchers due to its convenient operation and simple equipment [14,18,22,46]. TIPS is a fabrication method that does not need any specific device for extrusion or non-woven procedures. Besides, to simplify the scaffold fabrication, other processes such as in situ crosslinking and synthesis can be combined with the TIPS [236,261].

**Figure 11:**
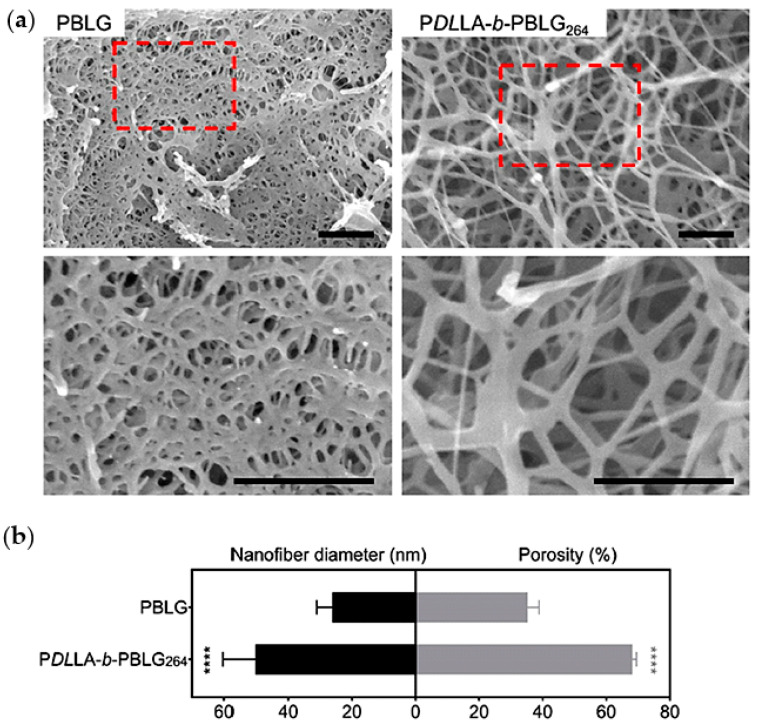
(**a**) SEM images of PBLG and PDLLA-*b*-PBLG 3D-scaffolds. Scale bars = 500 nm. (**b**) Quantification of nanofibre diameter and porosity in PBLG and PDLLA-b-PBLG scaffolds. **** *p* < 0.0001, *n* = 20 and 3 for nanofibre diameter and porosity analyses, respectively. Reproduced with permission from [68].

Liquid–liquid phase separation followed by nucleation and crystallization growth in the polymer-rich phase is an optimal procedure for the formation of nanofibres [262]. An alternative approach to form artificial 3D nanofibrous structures [263], is to microcrystallize a semi-crystalline polymer like PLLA through TIPS [264]. Using synthetic copolymers, Ma et al. developed nanofibrous scaffolds (50–500 nm) based on poly(hydroxyalkyl methacrylate)-*graft*-poly(L-lactide) (PHAA-g-PLLA) through TIPS [265,266]. In another attempt to produce an ECM-mimicking nanofibrous scaffold, Zhang et al. synthesized poly(d,l-lactide)-*b*-poly(γ-benzyl-l-glutamate) (PDLLA-*b*-PBLG), which underwent nanofibrous self-assembly by α-helix formation of the PBLG segment during controlled TIPS process (Figure 11). The derived scaffold showed a higher degradation rate, nanofibre size and porosity than scaffolds derived from neat PBLG. In addition, new scaffolds were highly cytocompatible and conducive to cell adhesion and proliferation. Due to facilitating neuron-like differentiation of seeded neural stem cells, this nanofibrous scaffold showed a great potential for neural tissue engineering [68].

**Figure 12:**
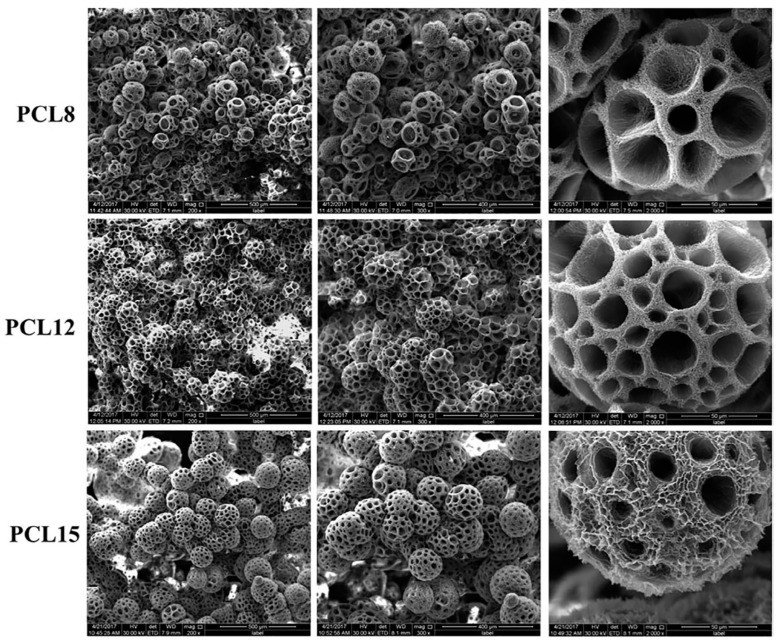
SEM images of PCL scaffolds obtained from 8, 12 and 15 *w*/*v* % of PCL in 1,4-dioxane/water (9:1) mixture, designated respectively as (PCL8), (PCL12) and (PCL15). Reprinted with permission from [18].

Recently, some researchers showed the possibility of producing microspheres-aggregated 3D porous scaffold by employing phase-separation and self-assembly processes without using any porogen agent or particular equipment [267,268,269]. Although their approach was intriguing, the resulting scaffold was lacking in some important properties for potential application in bone tissue engineering, for example the structure was not tunable and had low mechanical properties. In order to overcome these problems, Yao et al. [18] used the TIPS technique and, without the use of porogen agents, fabricated porous microspheres-aggregated PCL scaffolds with macropores, micropores, and nanofibrous-like structures that were suitable for bone tissue engineering. Dioxane:water (9:1) was selected as the solvent-nonsolvent system. Phase separation occurred by lowering temperature up to 273 K, and a nanofibrous structure was formed. In this case, a nonsolvent-induced phase separation also occurred throughout cooling. In addition, dioxane with the crystallization point of 288.8 K solidified during cooling, resulting in formation of a macro-porous structure upon the solvent evaporation. A spherulitic structure was also reported, which possibly could be ascribed to the growth of PCL spherocrystals during the TIPS process. Porosity, pore size, and mechanical properties of the scaffolds could in addition be controlled by a simple change of PCL concentration (Figure 12). High surface area of these constructs improved bioactivity, allowed a sustained release of multiple osteoinductive drugs, and showed high mechanical properties and an improved BMP2-induced osteogenic differentiation [18].

**Figure 13:**
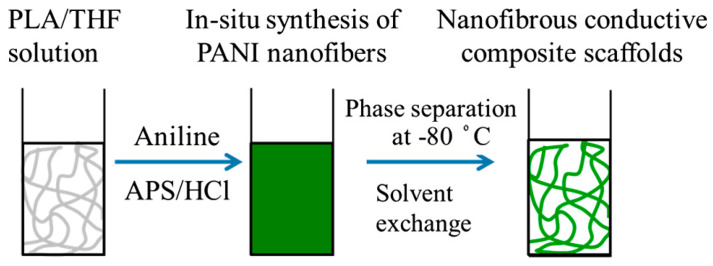
Schematic illustration of the process used for fabrication of conductive nanofibrous PLA scaffolds. Reproduced with permission from [67].

**Figure 14:**
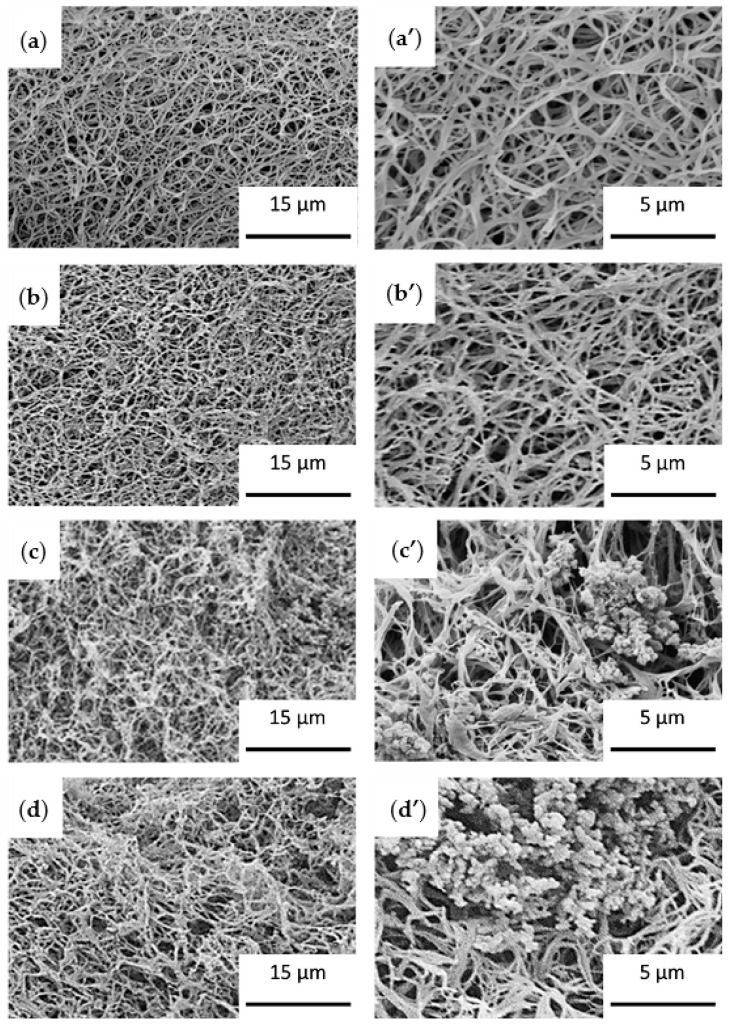
SEM micrographs of nanofibrous conductive scaffolds based on PLA and different PANI contents: 0 (**a**,**a’**), 15 (**b**,**b’**), 10 (**c**,**c’**) and 15 (**d**,**d’**). Reproduced with permission from [67].

Chen et al. [67] also made an interesting attempt to develop PLA nanofibrous matrices incorporating PANI nanostructures by combining in-situ polymerization and TIPS technique (Figure 13). The logic behind choosing conductive PANI was to overcome some PLA deficiencies like lack of chemical reactivity and biological inertness, which limit its scaffolding application when specific cell-material interactions are needed [270,271]. In-situ polymerization of conductive PANI nanoparticles in a PLA/THF solution followed by TIPS led to a 3D nanofibrous matrix (Figure 14) with interesting properties. Specifically, conductivity and electrochemical properties were evaluated for different PANI contents. Nanofibrous scaffolds with a moderate content of PANI significantly promoted osteogenic differentiation of bone marrow derived MSCs and were highly promising biomaterials for bone tissue regeneration [67].

Another interesting work concerning functionalization has been proposed by Guo et al. where an aminolysis process have been combined with TIPS to obtain PLLA nanofibrous scaffolds modified with poly(ethylenimine) (PEI). The ammonolytic agent, (i.e., PEI dispersed in dioxane) was dropwise added into PLLA/dioxane solutions to perform a typical TIPS process. The aminolysis reaction led to covalent bonds between amino groups of PEI and the ester groups of PLLA. Experimental conditions (i.e., polymer and modifier concentration, aminonolysis time and gelation temperature) affected the formation tendency, morphology of nanofibres and scaffold properties. Optimized conditions rendered a homogenous nanofibrous structure with high hydrophilicity, appropriate mechanical properties, high capacity to favour osteoblast proliferation and a great bone-bioactivity (i.e., apatite formation) [13].

### 5.2. Anisotropic Structures Prepared by Solid-Liquid Phase Separation

Physiological and mechanical properties of many tissues, such as tendon, nerve, blood vessel, bone, ligament, spinal cord and cartilage, are closely associated with their oriented structure [272]. Accordingly, the scaffolds used in tissue engineering and regenerative medicine can be prepared with an oriented-pore structure to better mimic these environments in vivo and guide the spatial cell organization during the tissue formation process as well as facilitating cell proliferation, migration and differentiation [273,274]. As an example, structure anisotropy is important for an ideal cardiac patch to mimic the morphologies of the native myocardium according to the alignment of elongated cardiomyocytes. Such anisotropic structure also allows the biodegradable patch to mechanically match the native myocardium. Mechanical mismatch between the biodegradable cardiac patch and the native myocardium may cause abnormal cardiac functions resulting in implantation failure [275,276]. Table 2 provides a summary of anisotropic/oriented scaffolds being recently fabricated by TIPS technique. Current relevant investigations and advances have been elaborated subsequently.

**Figure 15:**
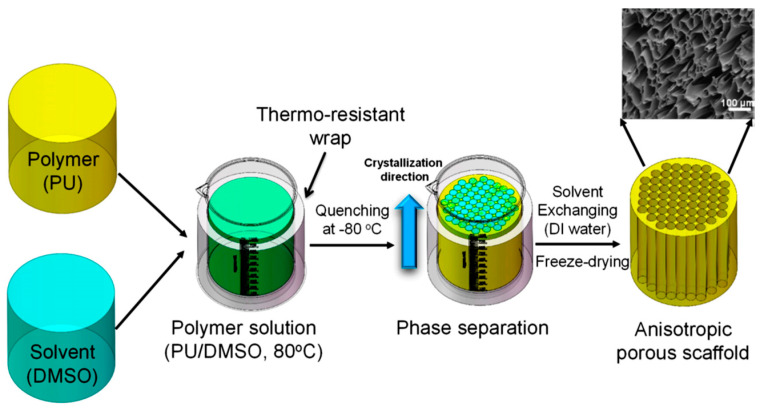
TIPS process for fabrication of anisotropic polyurethane porous scaffolds. Polyurethane was dissolved in DMSO at 80 °C and then directly quenched to −80 °C. The solvent crystallized in the uniaxial direction, was exchanged in deionized (DI) water and then lyophilized to produce anisotropic porous. Reprinted with permission from [71].

**Figure 16:**
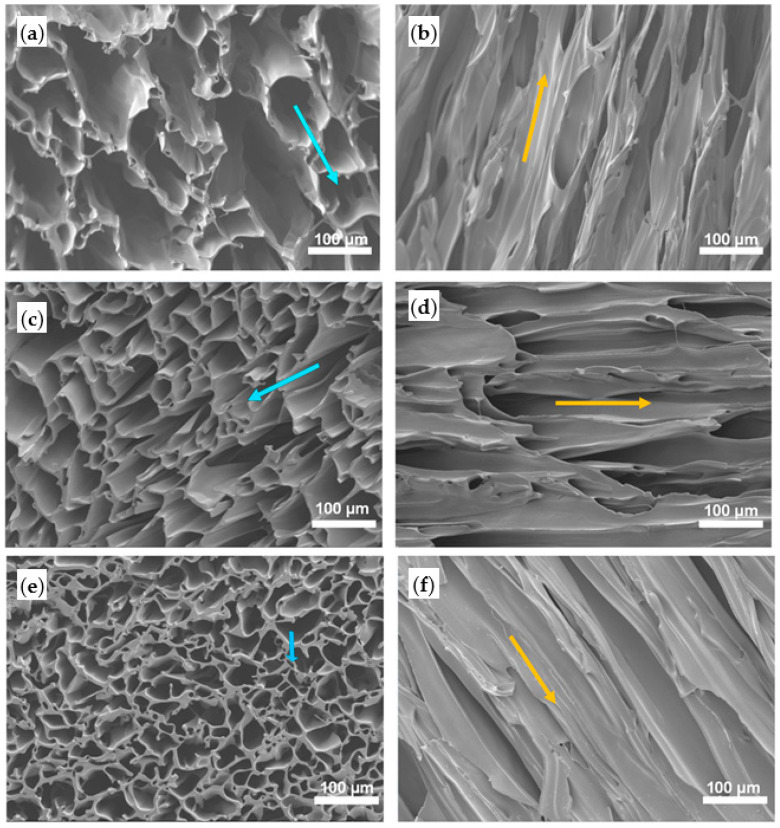
SEM micrographs of anisotropic porous polyurethane scaffolds prepared at −80 °C quenching temperature, from 5 (**a**,**b**)**,** 8 (**c**,**d**) and 10 (**e**,**f**) *w*/*v*% solutions. Micrographs correspond to transversal (**a**,**c**,**e**) and longitudinal (**b**,**d**,**f**) views are indicated by blue and yellow arrows, respectively. Reprinted with permission from [71].

To simulate the anisotropic architecture of native heart muscle, Xu et al. [71], by employing the TIPS technique, fabricated a series of anisotropic scaffolds made of biodegradable polyurethane to be used as cardiac patch. The synthetic polyurethanes with 5, 8 and 10 *w*/*v*% concentrations were dissolved in DMSO while a thermal-resistance wrap was employed to control crystallization in a uniaxial direction during the TIPS process (Figure 15). After solvent exchange with deionized water and freeze-drying, anisotropic scaffolds with the pores oriented along the crystallization direction were obtained. Honeycomb-like and aligned pore structures were observed in the transversal and longitudinal directions, respectively (Figure 16). The average pore sizes and porosities of the scaffolds decreased with increasing the polymer concentration (i.e., from 91 μm and 96% to 51 μm and 90%, for the scaffolds prepared from 5 and 10 *w*/*v*% polymer solutions, respectively). Mechanical properties of the anisotropic scaffolds were also optimized by altering polyurethane types and concentrations to match properties of the native human myocardium. The prepared biohybrid scaffolds were subsequently combined with decellularized and digested porcine myocardium-derived extracellular ECM to improve biocompatibility without affecting mechanical properties. The biohybrid scaffolds had morphologies similar to the acellular porcine myocardial matrix with good tissue compatibility and a minimal immune system response when they were subcutaneously implanted in rats [71].

Scaffolds with an oriented porous architecture can significantly promote cell infiltration and bioactive interflow between neo-host tissues [62,72]. Different examples can be found about in situ regeneration of osteochondral defects. Thus, by controlling the temperature gradient in radial or longitudinal directions throughout TIPS process, bioactive scaffolds with radially- or axially-aligned pore architectures have been developed to enhance in vitro/vivo cell migration and infiltration and promote tissue regeneration [274,277,278].

**Figure 17:**
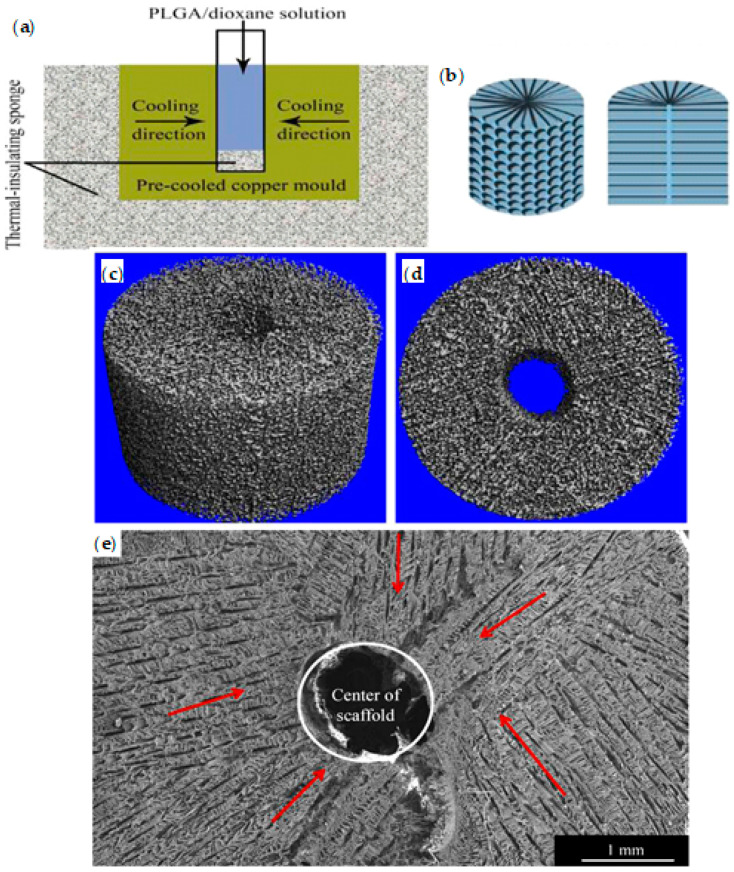
Schematic illustration of fabrication process (**a**) and the anticipated architecture (**b**) of radially-oriented PLGA scaffold through unidirectional cooling. Micro-CT topology evaluation; isometric (**c**) and top view (**d**) and SEM top view image (**e**) of the PLGA scaffold. The direction of oriented microtubules is shown by red arrows. Reprinted with permission from [62].

Recently, Dai et al. [279] have fabricated PLGA scaffolds with a radially-aligned structure through unidirectional cooling of polymer solutions in the radial direction for in vivo regeneration of osteochondral defects. By adding PLGA/dioxane solution into a polyethylene tube with a thermo-insulating sponge attached to the bottom of that (to prevent heat exchange from the axis direction), and by placing the tube in the central hole of a pre-cooled copper mould as a cooling bath, the cooling direction could be controlled and therefore the crystallization of dioxane along the radial direction. After lyophilization, a highly porous (>90%) cylindrical scaffold with radially-oriented microtubular pores perpendicular to the axial direction of the scaffold was obtained (Figure 17).

This construct enabled deep migration of bone marrow stem cells and could induce regular cell and ECMs alignment. It was also reported a simultaneous formation of cartilage and subchondral bone layers integrated with each other, while the smooth neo-tissues were well-integrated with the surrounding host tissues. Regeneration results were demonstrated to be much better than those found using PLGA scaffolds with random pores after the same implantation period, indicating the oriented-pore PLGA scaffold is a promising material for osteochondral regeneration [62]. Using natural resource polymers, Chen et al. also produced Col scaffolds with a radially-oriented pore structure, which could further improve the migration of bone marrow stem cells and regeneration of osteochondral defects compared to randomly-oriented scaffolds [279].

**Figure 18:**
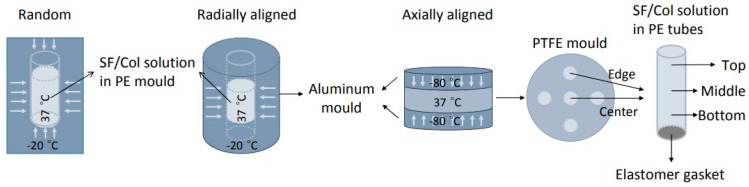
Schematic illustration of mould structures used for preparation of random, radially-aligned and axially-aligned SF/Col composite scaffolds. For fabricating the axially-aligned scaffold, polyethylene (PE) moulds containing the mixed solution was mounted into the holes of a larger PTFE mould, placed between two precooled (−80 °C) aluminum moulds to ensure an axially-aligned heat transfer. The white arrows indicate cooling directions. Reprinted with permission from [72].

**Figure 19:**
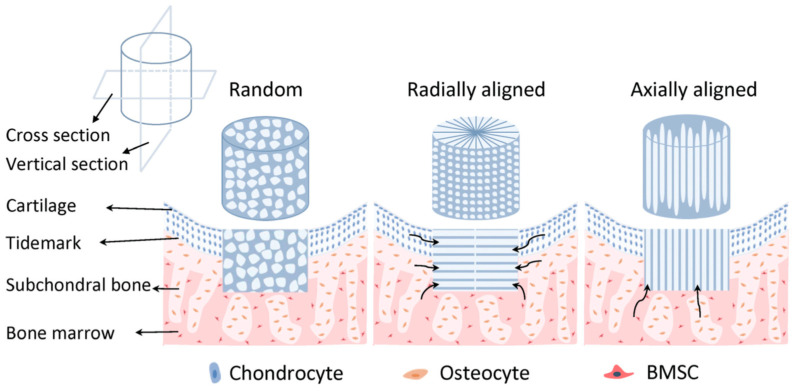
Schematic illustration of SF/Col composite scaffolds structures with random, radially-aligned and axially-aligned pores, and the implantation mechanism into osteochondral defects in vivo. Curvilinear arrows show how the radially-aligned and axially-aligned structures facilitate endogenous cell migration. Reprinted with permission from [72].

ECM-mimetic scaffolds based on silk fibroin (SF) and Col with different pore structures, including random pores, radially-aligned and axially-aligned pores were recently prepared by Feng et al. [72] using temperature gradient-guided TIPS. The effect of pore architecture on cell migration in the regeneration process of osteochondral defects was evaluated. To produce axially-aligned scaffolds they mounted a polyethylene tube containing the mixture solution of SF/water and Col/acetic acid into the holes of a larger PTFE mould placed between two pre-cooled aluminum moulds. Radially-aligned scaffolds were obtained following a procedure similar to that reported by Dai et al. [62], but using in this case they used a pre-cooled aluminum mould as a cooling bath (Figure 18). The obtained porous scaffolds had structural and mechanical anisotropy, porosity of about 85%, proper elastic modulus, and could support cellular in vitro and in vivo activities [72]. In vivo cell migration and infiltration was clearly improved in the cross-sectional direction (for the radially-aligned scaffolds) and in the vertical-sectional direction (for the axially-aligned ones) (Figure 19).

Although a satisfactory regeneration of osteochondral tissue with hyaline cartilage formation was observed in both aligned scaffold groups, the regeneration was faster in the radially-aligned scaffold group. Formation of hybrid cartilage (hyaline/fibrocartilage) was reported for the randomly oriented porous structure. Finally, the aligned SF/Col scaffolds, especially the radially-aligned ones, provided a promising alternative for regeneration of osteochondral defects, with a considerable potential to be translated into medical devices [72].

### 5.3. Crystalline Structures Prepared by Crystallization Induced-Phase Separation

When, due to thermodynamic conditions, a semicrystalline polymer-solvent system involves driving forces for liquid-liquid phase separation and crystallization of the polymer, the major factor determining which mechanism occurs is the strength of polymer-solvent interactions. In case of strong interactions (small differences between interaction parameters), the solution undergoes solid-liquid phase separation via crystallization of polymer during cooling. When the interactions are weak (great differences between interaction parameter), the polymer concentration determines the mechanism. Lloyd et al. reported that at high polymer concentration (e.g., higher than 59 wt% in case of isotactic polypropylene/n,n-bis (2-hydroxyethyl) tallowamine system) solid-liquid phase separation occurs (i.e., polymer crystalizes) and controls the morphology of the foam. At lower polymer concentrations (e.g., lower than 59 wt% in the above-mentioned system) liquid-liquid phase separation occurs and subsequently polymer crystalizes and stabilizes the phase separated structure. In the latter case, liquid-liquid phase separation controls the morphology [89].

Although a concentrated polymer solution has been used to fabricate various foams and monoliths for applications like membrane formation, generally low-concentrated solutions are used for preparing TE scaffolds (to achieve high porosity). Accordingly, individual utilization of solid-liquid phase separation (via polymer crystallization) as the main mechanism for fabrication of TE scaffolds does not occur very often. Nevertheless, crystallization of the polymer is occasionally observed when solid-liquid (caused by solvent crystallization) and liquid-liquid phase separations have been employed for fabrication of TE scaffolds. In such conditions crystallization of the polymer stabilizes the phase-separated structure and sometimes its corresponding morphological trace is observed throughout the microstructure of phase-separated regions [46].

Kinetic is another factor that may limit crystallization of the polymer during the TIPS process, even if it is thermodynamically favoured. The kinetic of phase separation process determines whether or not, and also to what extent the thermodynamically favoured mechanism occurs [81]. Since TIPS is a non-equilibrium process, the effects of the practical cooling rates on the equilibrium phase diagram must be considered [89]. Cooling the system in the experimental conditions of TIPS permits supercooling, and the crystallization of polymer shifts to lower temperatures than its equilibrium crystallization point. Conversely, the cooling rate has a minor effect on the location binodal curve (liquid-liquid phase separation temperature and the degree of undercooling required for inducing rapid binodal decomposition is very low [81,89]).

**Figure 20:**
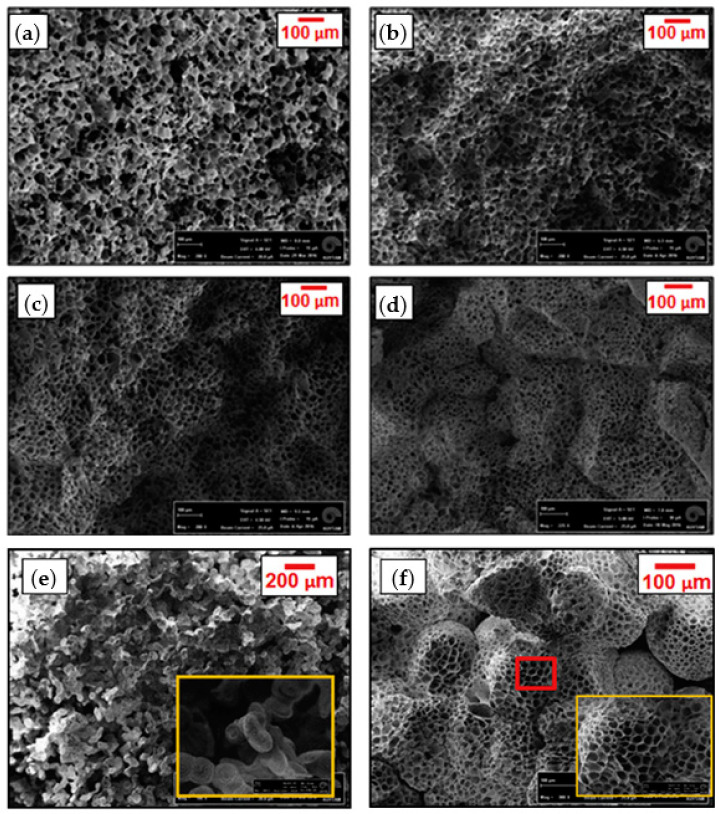
Cross-sectional SEM images of PLA foams prepared from a 8 wt% (**a**–**e**) and 10 wt% (**f**) PLA solution quenched to −20 °C (**a**–**d**), 24 °C (**e**) and 4 °C (**f**), and using a THF/water ratio of: (84/16) (**a**,**e**,**f**), (86/14) (**b**), (88/12) (**c**) and (90/10) (*w*/*w*) (**d**). Reprinted with permission from [90].

Onder et al. [90], by providing different experimental conditions for crystallization of polymer during liquid-liquid phase separation and the gelation process, prepared PLA foams by TIPS followed by solvent exchange and vacuum drying. Various ratios (84/16 to 90/10) of THF/water (solvent/nonsolvent) system were used. Phase separation was induced by cooling the PLA solutions (6–10 wt%) to three different temperatures (i.e., 24, 4 and −20 °C). The resultant PLA gels were subjected to solvent exchange (with ethanol) and then to vacuum-drying to achieve the porous, rigid and highly crystalline micro- and nano-structures. The porosity (between 85.1 and 92.8%), pore size (25–400 µm), morphology and mechanical properties of these rigid PLA foams was controlled by tuning the TIPS process parameters (i.e., polymer concentration, THF/water ratio and quenching temperature). The decrease of the water ratio in the binary solvent/nonsolvent system led to a gradual morphological change from homogeneous microcellular structures to microporous bead-like ones with smaller pore size (Figure 20a–d) and also to an increased crystallinity of the PLA foams. Upon cooling, the system first passed through the metastable region (NG mechanism) and then reached the unstable region (SD mechanism). The decreasing of water content in the solvent/nonsolvent system raised the power of the solvent system and the boundary of liquid-liquid phase separation (binodal curve) shifted to a lower temperature. Accordingly, the system will stay longer in the metastable region and shorter in the unstable region. In such a condition, the polymer had enough time to crystallize through NG mechanism and formed to a greater extent the bead-like morphology (arising from NG of polymer crystals at relatively low concentrations) than the fine cellular morphology (arising from SD mechanism). This effect was more significant upon slower cooling (i.e., higher quenching temperature) (Figure 20e,f) [90].

**Figure 21:**
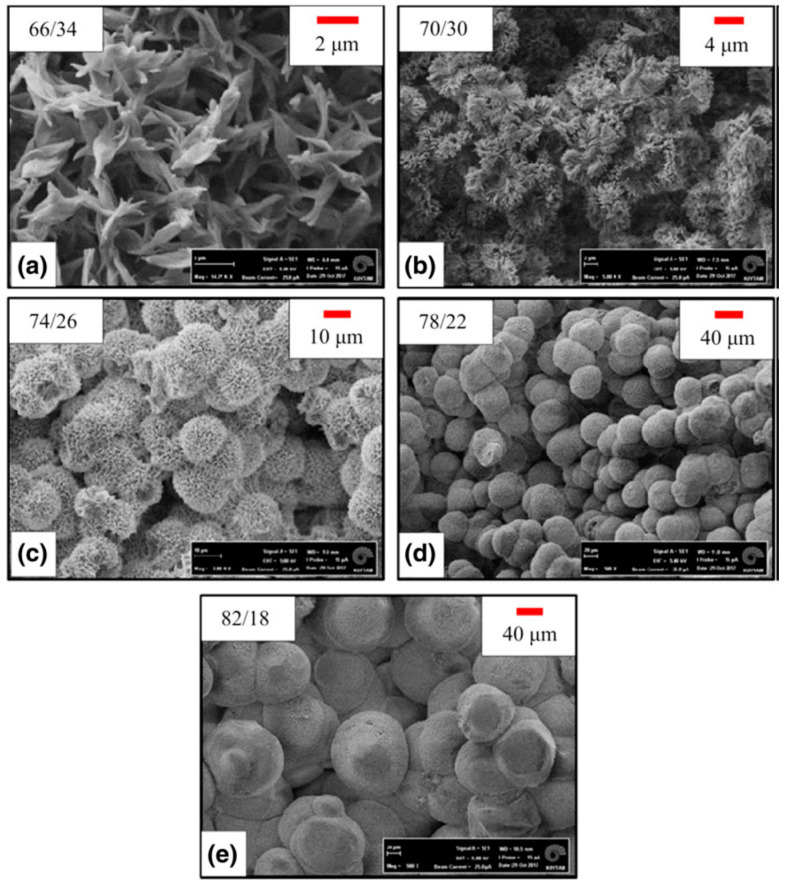
Change in the morphologies of PLA foams obtained through polymer crystallization mechanism as a function of THF/methanol ratio from 66/34 to 82/18 (**a**–**e**), at 18 wt% polymer concentration. Reprinted with permission from [14].

In another recent study, Onder et al. have developed PLA foams with various architectures and morphologies from various ratios of THF/methanol (solvent/nonsolvent) after inducing phase separation/gelation by quenching. Resultant gels were stabilized by exchanging the solvent system with ethanol and porous scaffolds were obtained after quenching and supercritical CO_2_ drying. Crystallinity, porosity, pore size and structure could be controlled by altering two TIPS processing parameter (i.e., polymer concentration from 8 to 22 wt%) and solvent system quality (i.e., solvent/nonsolvent ratio from 56/44 to 82/18) (Figure 21). The different architectures arisen from polymer crystallization or from SD mechanism during the TIPS process. Higher crystallinity of PLA foams with the orthorhombic α-form crystal structure was reported at lower polymer concentrations and higher THF solvent contents. In such conditions, various crystalline morphologies like thin lamellar platelets, lamellar stacks, axialites and spherulites were observed. High methanol nonsolvent contents led always to interconnected 3D polymer networks arising from SD mechanism. A decrease in average pore size and the porosity of the 3D polymer foams was also reported at higher PLA concentrations [14].

**Figure 22:**
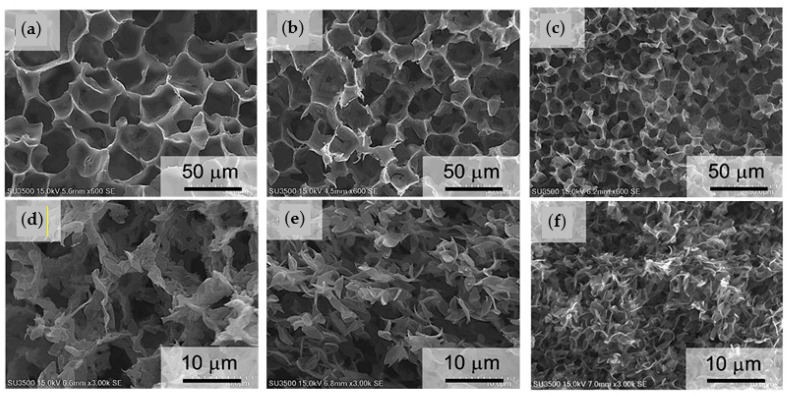
SEM images of PLLA monoliths obtained from different ratios of 1,4-dioxane/2-butanone/water: (**a**) 85/0/15, (**b**) 65/20/15, (**c**) 45/40/15, (**d**) 25/60/15, (**e**) 20/70/10, (**f**) 15/80/5. Reprinted with permission from [281].

PLA monoliths are suitable to be used in several fields, such as tissue engineering, drug delivery and filtration technology. These monoliths have been produced using TIPS methods based on binary solvent systems of THF-water [90] and 1,4-dioxane-water [280]. Monoliths showed relatively large pores (25–400 µm) and short-range micron-scale frameworks (10–30 µm), respectively. PLA monoliths have also recently been developed by Kanno and Uyama [281] using a novel TIPS technology. A ternary system including 1,4-dioxane, water and 2-butanone was used as good solvent, non-solvent and midsolvent of PLA, respectively. Using this ternary system, the phase separation process was efficiently controlled and leaf-like morphologies with frameworks in a wide-range scale (i.e., from micron to nano) were produced. By modifying only the solvent ratios a precise control on the morphology and foam characteristics could be achieved. As a result, monoliths with a pore size of 1.1–28.6 µm, skeletal size of 200–2500 nm and porosity around 90–93% were obtained. The novelty of this investigation was the addition of a midsolvent (e.g., 2-butanone), which in addition was able to promote a slow TIPS process and provide sufficient time for PLLA to crystallize (i.e., final crystallinity could be increased from 45 to 66.5%). In such condition, a transition from wall-like morphologies (arising from liquid-liquid phase separation) to interconnected leaf-like structure (obtained via crystallization of the polymer) was also observed (Figure 22) [281].

## 6. Conclusions

Porous biodegradable scaffolds play a critical role in tissue engineering. Scaffolds, by mimicking the native ECM, provide physical bio-templates for cells, allowing them to attach, proliferate and guide the formation of new tissues. TIPS is one of the scaffold fabrication methodologies with a great potential for production of highly porous scaffolds with a wide range of pore size/architecture and a proper structural interconnectivity. These characteristics, together with a simple, low-cost and tunable fabrication process, which allows the scientist to incorporate various fillers and/or bioactive agents, have made it a popular method for fabricating polymeric and composite scaffolds. The process enables the generation of a variety of structures with different properties being desirable for regeneration of tissues using specific target cells. Although TIPS is a conventional method for preparing tissue engineering scaffolds, researchers have successfully innovated new approaches in terms of biomaterial selection and variations on the processing parameters, being enhanced its capability and versatility. Herein we reviewed the latest innovations and the recent advancements in scaffolds fabricated by the TIPS technique from the viewpoint of methodology, materials selection and structural features. Owing to the outstanding potentials and promising achievements of this method in producing tissue engineering scaffolds, conducting more investigations and up-to-date surveys in this field seems inevitable.

## Figures and Tables

**Table 1:** Summary of some scaffolds fabricated recently by combination of TIPS and other techniques.

Polymer System	Filler System	Solvent System	Morphology	Fabrication Technique	Application	Reference
PLLA	HA	Dioxane	Microporous	TIPS + salt leaching	BONE TE	[60]
PLGA		Dioxane	Radially-aligned and random pores ^1^	TIPS + salt leaching	OSTEOCHONDRAL	[62]
GEL	Nanosilicate	Ethanol:water (1:1)	Nanofibrous	TIPS + porogen leaching ^2^	BONE TE	[61]
GEL/PEEUU ^3^	MSN/SA ^4^	Water/HFIP ^5^	Microporous and nanofibrous ^6^	TIPS + electrospinning	VASCULAR TE	[58]
PCL/Col	CNFs	Dioxane/acetic acid ^7^	Microporous and nanofibrous ^8^	TIPS + electrospinning	NEURAL TE	[59]
PLLA/CS	PDA/Qu ^9^	Acetic acid	Microfibrous and nanofibrous ^10^	TIPS + 3D printing	BONE TE	[63]
PLGA	nHA ^11^	Dioxane	Microporous and macroporous ^12^	TIPS + 3D printing	BONE TE	[22]
PLGA		EC	Microporous and knitted mesh ^13^	TIPS + textile technology	DERMAL TE	[20]

^1^ Radially-aligned pores were obtained from TIPS, random pores from salt leaching; ^2^ Paraffin spheres have been used as porogen; ^3^ Poly(ester-urethane)urea (PEEUU); ^4^ Mesoporous silica nanoparticles (MSNs) loaded with salvianic acid (SA); ^5^ Distilled water was used as solvent for Gel/MSN/SA system (in TIPS), hexafluoro-2-propanol (HFIP) for PEEUU (in electrospinning); ^6,8^ The scaffolds have a bilayered (microporous/nanofibrous) tubular structure; ^7^ 1,4-dioxane for PCL/CNFs system (in TIPS), acetic acid for PCL and Col (in electrospinning); ^9^ polydopamine (PDA), quercetin (Qu); ^10,12^ The scaffolds have a hierarchical structure; ^11^ nanohydroxyapatite (nHA); ^13^ The scaffold has a biphasic structure.

**Table 2:** Summarized information of some scaffolds fabricated recently by the TIPS technique.

Polymer System	Solvent System	Structure	Fabrication Technique	Porosity	Application	Reference
PLLA ^1^	1,4-dioxane	Anisotropic pores	TIPS	~91–92%	Bone TE	[73]
polyurethane	DMSO	Anisotropic pores	TIPS (controlled heat direction)	~93–97%	Cardiac patch	[71]
PLLGC	1,4-dioxane	Oriented microtubular	TIPS	~73–85%	TE	[25]
SF ^2^/Col	water/acetic acid ^3^	radially/axially- aligned pores	TIPS (temperature gradient-guided)	~85%	Osteochondral	[72]
PHBV ^4^	1,4-dioxane	Oriented pores, unidirectional channels	TIPS	~41–77%	TE	[74]
PEUU	DMSO	Oriented connected macropores	TIPS		Rotator cuff repair	[66]
PLLA	1,4-dioxane ^5^	Anisotropic pores, (~parallelepiped)	TIPS (solid-liquid phase separation)	~75–95%	TE	[64]
PLGA	1,4-dioxane	Radially-aligned pores	TIPS (uniaxial temperature gradient)	~91%	Osteochondral	[62]

^1^ The solution incorporated TiO_2_ nanoparticles functionalized with OA; ^2^ silk fibroin (SF); ^3^ Water as solvent for SF, acetic acid for Col; ^4^ The solution incorporated CNCs; ^5^ In case of dioxane/water as solvent, the microstructure was controlled by liquid-liquid phase separation and an anisotropic structure was not observed.

## Data Availability

No new data were created or analyzed in this study. Data sharing is not applicable to this article.

## Abbreviations

3D	Three-dimensional
Alg	Alginate
AM	Additive manufacturing
β-TCP	Beta-tricalcium phosphate
BG	Bioactive glass
CaSi	Calcium silicate
CNC	Cellulose nanocrystals
Col	Collagen
Col1	Collagen type 1
CNFs	Carbon nanofibres
DCPD	Dicalcium phosphate dihydrate
DMF	Dimethylformamide
EAB	Ethyl alcohol bath
ECM	Extracellular matrix
EV	Exosome vesicle
FDA	Food and Drug Administration
FESEM	Field emission scanning electron microscope
GEL	Gelatin
GelMA	Gelatin methacryloyl
HAc	Hyaluronic acid
hAD-MSC	Human adipose mesenchymal stem cell
HA	Hydroxyapatite
HFIP	Hexafluoro-2-propanol
HSP	Hansen Solubility Parameters
LCA	Life Cycle Assessment
LD50	Lethal dose
MicroCT	Micro-computed tomography
μFC	Micro-fluorcanasite
MSNs	Mesoporous silica nanoparticles
MWCNT	Multiwalled carbon nanotube
MSCs	Mesenchymal stem cells
NG	Nucleation and growth
NGC	Neural guidance channel
nHA	Nanohydroxyapatite
OA	Oleic acid
PANI	Polyaniline
PCL	Polycaprolactone
PDA	Polydopamine
PDLLA	Poly(d,l-lactide)
PDLLA-*b*-PBLG	Poly(d,l-lactide)-*b*-poly(γ-benzyl-l-glutamate)
PEG	Polyethylene glycol
PEI	Poly(ethylenimine)
PEEUU	Poly(urethane urea)ester
PGA	Polyglycolide
PGCL	Poly-(glycolide-*co*-caprolactone)
PHAA-g-PLLA	Poly(hydroxyalkyl methacrylate)-graft-poly(l-lactide)
PHB	Poly(3-hydroxybutyrate)
PHBV	Poly(3-hydroxybutyrate-*co*-3-hydroxyvalerate)
PLA	Polylactide
PLCL	Poly(lactide-*co*-caprolactone)
PLGA	Poly(lactide-*co*-glycolide)
PLLA	Poly(l-lactide)
PLLGC	Poly(l-lactide-*co*-glycolide-*co*-ɛ-caprolactone)
PTFE	Polytetrafluoroethylene
Qu	Quercetin
RBMSCs	Rabbit bone mesenchymal stem cells
SA	Salvianic acid
SC-CO_2_	Supercritical carbon dioxide
SD	Spinodal decomposition
SEM	Scanning electron microscopy
TCH	Tetracycline hydrochloride
TCP	Tricalcium phosphate
TE	Tissue engineering
THF	Tetrahydrofuran
TIPS	Thermally-induced phase separation
TWB	Thermal water bath
UCST	Upper critical solution temperature
